# Comprehensive mapping of the virus and host factors that guide the paths of HIV-1 escape from a therapeutic

**DOI:** 10.1016/j.celrep.2026.117558

**Published:** 2026-06-19

**Authors:** Aaron N. Gillman, Cassian M. Birler, Rohith Rao Vujjini, Mohammad Fili, Samuel A. McCarthy-Potter, Wilson Chen, Madeline M. Broghammer, Guiping Hu, Margaret J. Gartland, Manyu Prakash, Annika Helverson, Grant G. Brown, Hillel Haim

**Affiliations:** 1 Department of Microbiology and Immunology, The University of Iowa, Iowa City, IA, USA; 2 Department of Systems Engineering and Operations Research, George Mason University, Fairfax, VA, USA; 3 ViiV Healthcare, Durham, NC, USA; 4 ViiV Healthcare, Brentford, UK; 5 Department of Biostatistics, School of Public Health, The University of Iowa, Iowa City, IA, USA; 6 These authors contributed equally; 7 Lead contact

## Abstract

HIV-1 resistance to therapeutics can emerge through diverse mutational routes, yet the determinants guiding pathway selection *in vivo* remain unclear. Through comprehensive screening, we identify 18 mutations in the HIV-1 Env protein that enhance resistance to the FDA-approved small-molecule therapeutic temsavir. We then examine their occurrence in HIV-infected individuals who developed resistance on therapy. Interestingly, only a subset of the resistance-enhancing mutations emerged *in vivo*. On-treatment mutation frequencies correlate with their emergence rates in temsavir-untreated individuals and are governed by two parameters: (1) probability of mutation appearance, determined by the number and type of nucleotide changes required, and (2) probability of mutation persistence, determined by Env functional and immune fitness. Notably, non-neutralizing antibodies commonly elicited in HIV-infected individuals restrict emergence of multiple resistant forms, driving convergence to a narrow set of escape routes. These findings establish a quantitative framework for predicting therapeutic resistance and reveal how host immunity constrains viral evolution during treatment.

## In brief

Gillman et al. systematically screen for HIV-1 Env mutations that confer resistance to the FDA-approved therapeutic temsavir (BMS-626529). They discovered that in treated individuals, resistance mutations converge to limited pathways determined by mutation probability, viral fitness, and conserved host immune pressures, providing a framework to predict evolutionary outcomes during therapy.

## Graphical abstract

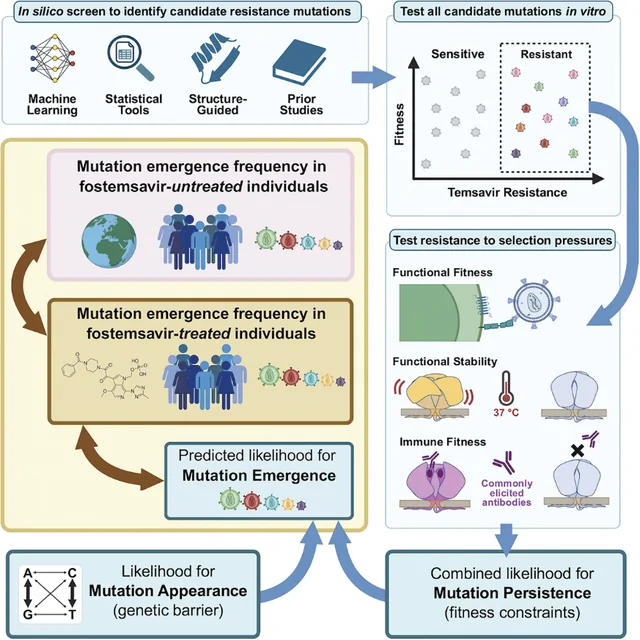

## INTRODUCTION

Human immunodeficiency virus type 1 (HIV-1) has a remarkable ability to adapt to the host environment. Due to the low fidelity of the viral replication machinery,^1,2^ nucleotide substitutions are introduced randomly across the genome, allowing the virus to sample new variants of its proteins and regulatory elements.^3,4^ Such changes facilitate the broadening of cell tropism,^5,6^ evasion of the host immune responses,^7,8^ and development of resistance to antiviral therapeutics.^9^ Clinical resistance can emerge by *de novo* mutations that occur during treatment or by resurfacing of archived forms from latent cell reservoirs.^10^ Given that therapeutics dock to their protein targets via interaction with multiple residues, several mutational pathways are available for the virus to evade recognition. However, as most antiretrovirals target sites associated with protein function, resistance-enhancing mutations (REMs) often compromise virus fitness, limiting the ability of these variants to persist in the host. In such cases, adaptive mutations that increase fitness yet retain the resistance phenotype can facilitate persistence.^11–13^

While functional fitness and inhibitor resistance are clearly traits essential for virus replication, the principles that govern the selection of escape paths from therapeutics remain incompletely understood. First, there is a need for a more quantitative understanding of the contributions of these two traits. For example, is a modestly resistant but highly fit variant more likely to persist than a highly resistant but poorly fit form, and what thresholds define these distinctions? Second, to what extent do factors other than functional fitness and inhibitor resistance contribute to the appearance and persistence of the new variants? For example, amino acid substitutions that require a single-nucleotide change are more likely than those requiring two changes. However, the likelihoods for all nucleotide changes, at least *in vitro*, are not identical, with transitions exhibiting higher rates than transversions.^14–16^ Whether such preferences influence escape pathway selection *in vivo* is unknown. Third, the extent to which we can identify all possible mutational paths that impart resistance to a therapeutic is still unclear. Indeed, HIV-1 proteins, and particularly the envelope glycoproteins (Envs), exhibit considerable within- and between-host variability.^17,18^ Such variability results in strain-specific conformations and allosteric effects. Consequently, some mutations may increase resistance or reduce fitness of some isolates but exert limited effects on others. Context dependence of mutation effects may limit our ability to estimate the resistance of HIV-1 isolates from sequence data, reducing the potential for personalized antiviral therapies. Context-specific effects may also hinder the generalization of *in vitro* findings to the diverse viruses that circulate in the population.

To address these questions, we investigated the *in vivo* escape paths of HIV-1 from the Env-targeting inhibitor temsavir (TMR, BMS-626529), the active metabolite of the FDA-approved prodrug fostemsavir.^19,20^ TMR effectively reduces viral loads in HIV-infected individuals.^21–23^ In addition, it has been shown to reduce the proinflammatory effects of soluble gp120 and to prevent killing of uninfected CD4-positive cells via effector-mediated mechanisms.^24–26^ We first conducted a comprehensive effort to identify the mutations that increase HIV-1 resistance to TMR *in vitro*. Then, we examined their representation in participants of the BRIGHTE clinical trial who developed resistance to this agent during treatment.^21,22,27^ Interestingly, only a subset of the REMs identified *in vitro* emerged on treatment. REM emergence frequencies correlated strongly with their spontaneous emergence rates in the fostemsavir-untreated population. *In vitro* analysis of the functional and antigenic properties of all REMs as well as *in silico* modeling of their substitution likelihoods revealed that their rates of emergence *in vivo* were explained well by these parameters. Remarkably, non-neutralizing antibodies against Env, which are commonly produced in HIV-infected individuals, appeared to restrict the range of resistance mutations that emerged *in vivo*. Together, our findings establish a quantitative framework for understanding the emergence of resistance to antiviral therapeutics and inform strategies to predict the rate and paths of escape in each individual.

## RESULTS

### Mutations in the core epitope of TMR account for most, but not all, resistance to TMR

**Figure 1.**
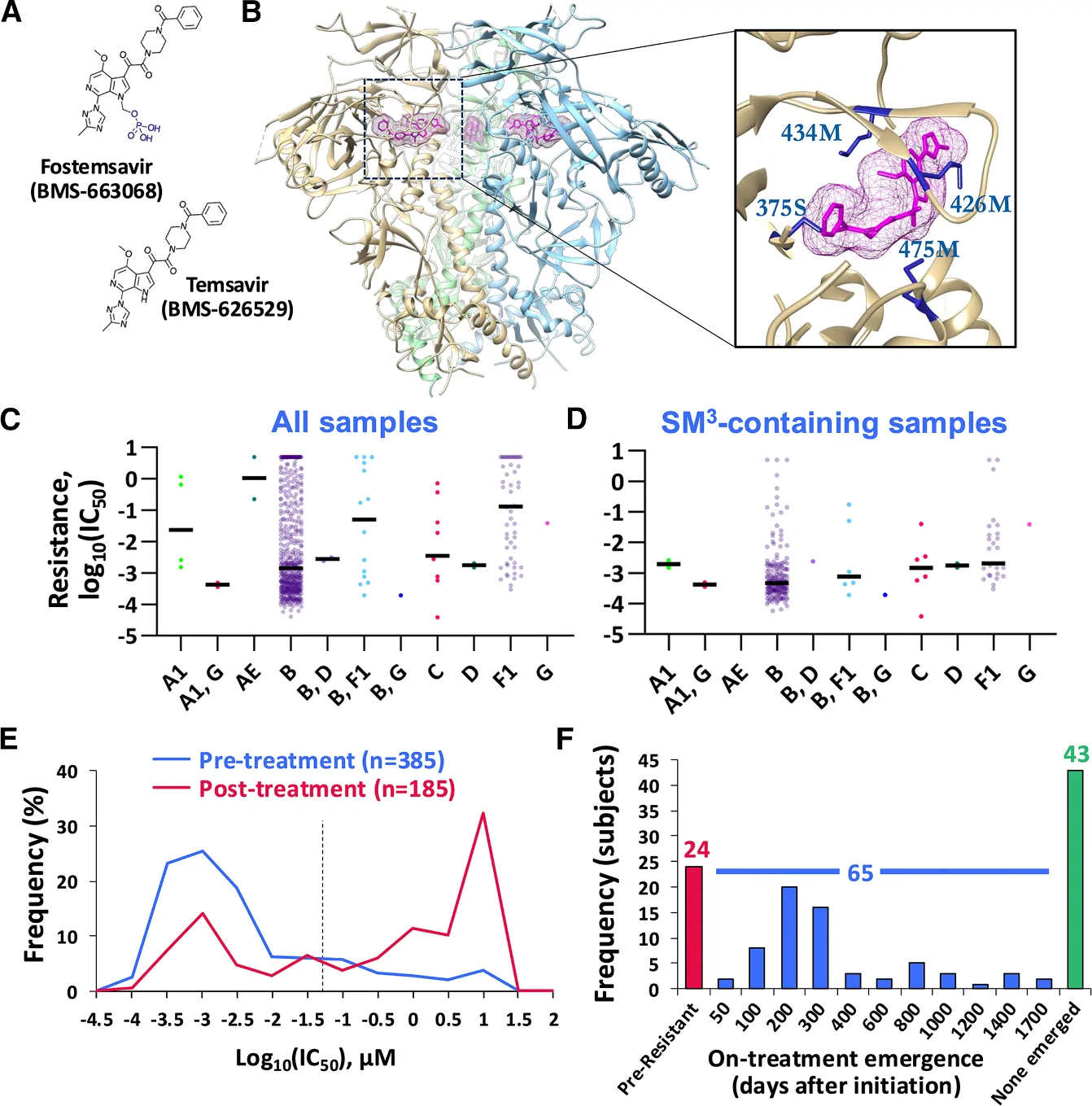
TMR resistance and escape in the BRIGHTE clinical trial (A) Structure of fostemsavir and the active metabolite TMR. (B) Cryo-electron microscopy model of the Env trimer bound to TMR (in magenta, PDB ID 8TTW). The inset shows the CD4-binding pocket with the SM^3^ sites labeled. (C and D) Resistance values of all 570 samples from BRIGHTE trial participants and of the subset of samples that contains the TMR-sensitive SM^3^ motif. Samples are grouped by their inferred clade associations. (E) Distribution of IC_50_ values for samples collected before and after fostemsavir treatment. The threshold used to define resistance (50 nM) is shown by a dotted line. (F) TMR resistance outcomes in the 132 BRIGHTE trial participants for whom genotype and IC_50_ data were available for samples collected both before and after treatment.

The small-molecule inhibitor TMR is generated by hydrolysis of the orally administered prodrug fostemsavir in the gut^28–30^ (Figure 1A). TMR targets the CD4-binding pocket of Env and stabilizes the trimer in a CD4-unbound state, preventing activation of the entry cascade by the CD4 receptor^31^ (Figure 1B). The main contact sites for TMR are the side chains of Env positions 375, 426, and 434, which cradle this molecule in the pocket.^27,32–36^ Mutations at position 475 have also been shown to impact resistance.^36–39^ The consensus amino acid motif at these four sites for TMR-sensitive strains is Ser at position 375 and Met at positions 426, 434, and 475, collectively designated herein the SM^3^ motif. Mutations at these sites reduce virus sensitivity to TMR *in vitro* and were associated with clinical resistance in treated individuals.^22,27,32^ Several trials were conducted to test the efficacy of fostemsavir and predecessor compounds. The largest trial, BRIGHTE, enrolled 371 HIV-positive participants, who were treated for up to 5 years^21–23,26,27,40^ (see trial design in the STAR Methods section). Plasma samples were collected from the participants before and during treatment and analyzed by the Monogram Biosciences PhenoSense Entry assay combined with the HIV Envelope NGS Sequencing Assay. The assays are based on amplification of *env* from plasma virus and bulk cloning of the amplicons into an expression vector.^33,41^ The library of plasmids from each sample is used to generate libraries of pseudoviruses that are tested *in vitro* for resistance to TMR, as determined by the concentration required to achieve a 50% reduction of infection (IC_50_). In addition, amplicons from the samples were used to obtain sequence data for the entire *env* gene. The trial thus provided genomic and phenotypic data that we used to establish the escape paths of the virus *in vivo* (see Data S1).

Sequence and TMR IC_50_ data were available for 570 plasma samples from 360 BRIGHTE participants. We first examined the distribution of IC_50_ values based on their HIV-1 subtype associations (see phylogenetic tree in Figure S1A and Data S2). Of the 360 samples, 83.3% were identified as clade B, 3.5% as clade B recombinants, and 9.5% as clade F1. Considerable variability was observed in IC_50_ values within and between the clades (Figure 1C). As expected, CRF01_AE Envs were resistant to TMR due to the presence of the S375H mutation.^42^ Variability in IC_50_ values was significantly lower in the subgroup of samples that contained the SM^3^ motif (*p* value <10^−6^ in an F-test for equality of variances between the IC_50_ values in the two groups, compare Figures 1C and 1D). Similarly, variability was lower in the samples that contain the SM^3^ motif relative to those that do not contain it (*p* value <10^−6^). Nevertheless, some SM^3^-containing samples still exhibited high IC_50_ values, suggesting that additional sites impact resistance. We also performed the above analysis for a previously published panel of 208 Envs (designated herein the Single-Env dataset) that was tested for resistance to TMR.^38^ A similar decrease in the variability in IC_50_ values was observed for the SM^3^-containing Envs of this panel (Figure S1B and Data S3). This finding suggested that positions outside the SM^3^ motif may contribute to resistance.

To establish a threshold that distinguishes between sensitive and resistant samples, we examined the distribution of IC_50_ values for the BRIGHTE dataset. A right-skewed distribution was observed for the pre-treatment samples and a bimodal one for the post-treatment samples (Figure 1E). Based on these data, we selected an IC_50_ threshold of 50 nM TMR, which represents the 85^th^ percentile for the pre-treatment samples. This threshold was then used to define changes in resistance status during treatment. Among the 360 participants, only 132 had sequence and IC_50_ data for both pre- and post-treatment time points (Figure 1F). Of these, 24 were resistant to TMR before treatment, 43 remained sensitive to TMR throughout treatment, and 65 gained resistance on treatment (with a median time of 218 days). For this latter group, designated herein the escape group, we sought to identify and characterize the mutational paths used by the virus to gain resistance.

### A combined approach to identify Env mutations suspected of increasing HIV-1 resistance to TMR

**Figure 2.**
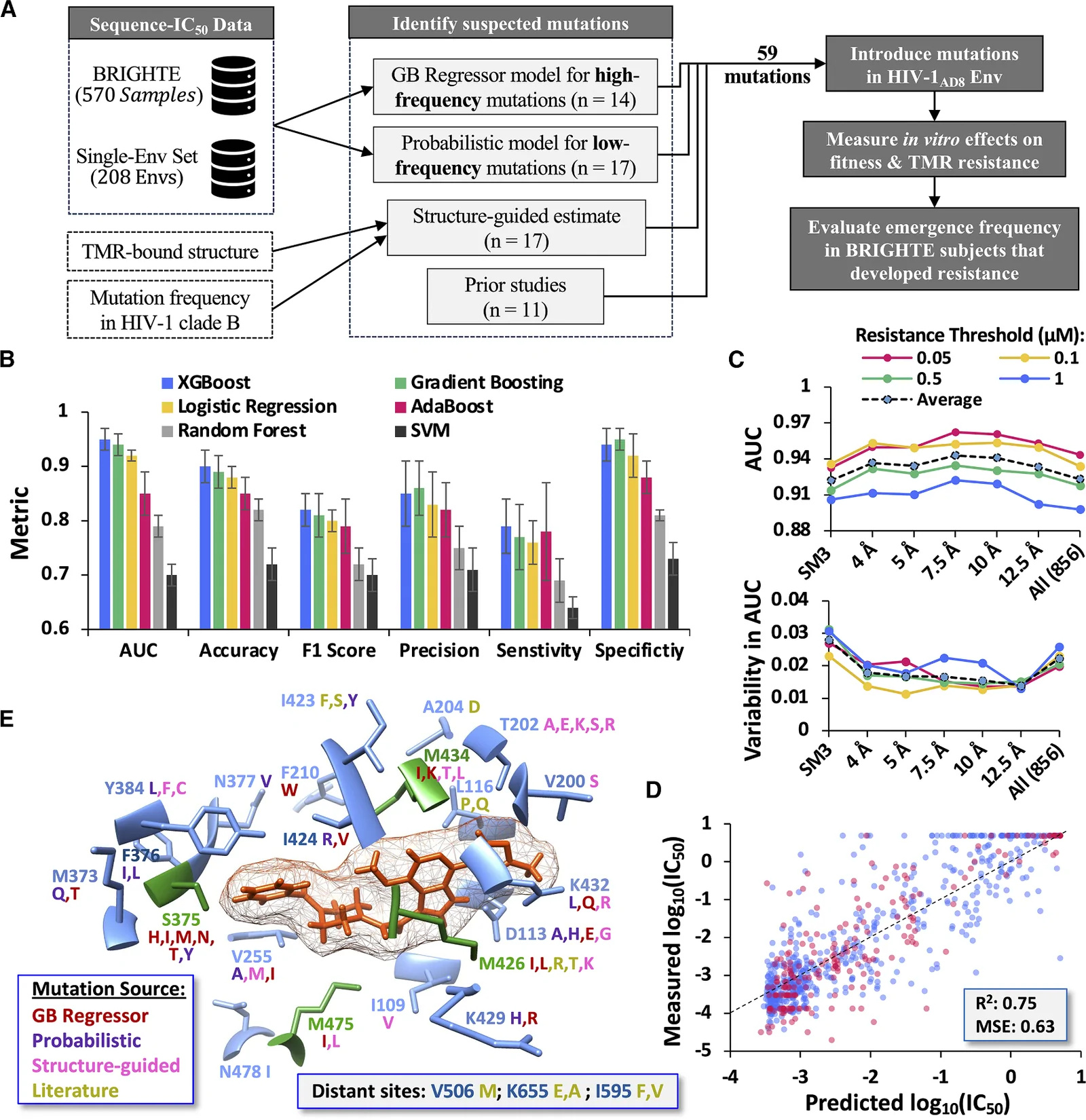
Identification of mutations suspected of increasing HIV-1 resistance to TMR (A) Our approach to identify the mutations. The number of mutations identified by each approach is shown. (B) Performance of different algorithms to predict TMR resistance by sequence. Amino acid sequence at the 856 positions of Env in the 570 BRIGHTE trial samples was used as input. Average metrics for 5-fold cross-validation are shown. Error bars, standard deviation (SD). (C) Performance of the XGBoost algorithm to predict TMR resistance in the BRIGHTE samples by Env sequence. As input, we used amino acids at the four SM^3^ positions, all 856 positions, or positions within the indicated distances from TMR on the TMR-bound structure of Env. Performance was tested using the indicated IC_50_ thresholds to define resistance. Average AUC values and their variability (SD) across the five folds are shown. (D) Performance of a Gradient Boosting Regressor model to predict resistance of 778 samples from the BRIGHTE (blue) and Single-Env (red) datasets by sequence. MSE, mean squared error. (E) The 59 Env mutations suspected of increasing resistance to TMR identified by the four approaches shown in (A).

To identify the escape paths from TMR in the BRIGHTE participants and compare them with all possible paths available to the virus, we pursued the approach described in Figure 2A. First, we used four strategies to identify Env mutations suspected of increasing resistance to TMR. We then tested them *in vitro* for their effects on TMR resistance and Env function using a pseudovirus infection assay. Finally, for the REMs identified, we examined their emergence frequencies after treatment in the escape group, to determine if the observed frequencies can be explained by their effects on fitness and/or TMR resistance.

To determine if the genotype-phenotype datasets from the BRIGHTE trial are sufficiently informative to identify mutations that increase resistance to TMR, we tested different machine learning models (see details in the STAR Methods). As input for the models, we used the amino acids at all 856 positions of Env according to the HXBc2 numbering system.^43^ The outcome to be predicted was the presence of resistance (IC_50_ value greater than 50 nM). The XGBoost and Gradient Boosting classifier algorithms^44^ achieved the highest performance across all key metrics (Figure 2B; Figure S2A) and were thus chosen as the foundations for subsequent models. We then evaluated the optimal number of Env positions to include as input, which were selected based on their minimal distance (in Å) from the TMR molecule using coordinates of the TMR-liganded Env^38^ (PDB ID 5U7O). Positions within 4, 5, 7.5, 10, or 12.5 Å from any TMR atom were tested (see positions in Table S1). In addition, we tested the four sites of the SM^3^ motif, and all 856 positions of Env. Figure 2C shows the area under the curve (AUC) metric for these tests, which describes the performance of the model to distinguish between resistant and sensitive samples by sequence, whereby a value of 1 indicates perfect discrimination and 0.5 corresponds to random classification. AUC values greater than 0.9 were observed for most sets, peaking at 0.96 for the 56 positions located within 7.5 Å of TMR (see all metrics in Figures S2B and S2C).

To validate the ability to accurately estimate resistance by sequence and broaden the clade representation, we expanded the dataset to include the 208 Single-Env dataset (see normalization of the IC_50_ values in Figure S3A). As expected, analysis of the combined dataset as well as training on the BRIGHTE data and testing on the Single-Env samples exhibited high performance (Figures S3B and S3C). Furthermore, the model generalized well across clades: training on clade B and testing on non-clade B samples resulted in only a modest decline in performance relative to the clade-agnostic model (Figure S3D).

Performance of the classification-based model was highest for Env positions within 7.5 Å of the TMR molecule (see also standard deviations across the five folds in Figure 2C). Furthermore, standard leave-one-out analysis did not identify elements outside the CD4-binding pocket that contribute to the prediction (Figures S3E and S3F). As such we selected the 7.5 Å subset for subsequent analyses to identify the specific mutations that impact resistance. To this end, we used a GB Regressor algorithm. The Shapley Additive Explanations (SHAP) value was used to quantify the contribution of mutations to the model’s predictive capacity.^45^ As shown in Figure 2D, performance of the GB Regressor model was high. Importantly, it provided a list of mutations, each with an estimated effect on resistance (Figures S4A and S4B). The 14 mutations with mean absolute SHAP values greater than 0.01 (Data S4) were defined as suspected of impacting resistance and were further tested *in vitro* as described below.

Machine learning algorithms identify mutations that are frequently sampled in the datasets. To identify mutations that are less frequently sampled, we used a probabilistic modeling approach that only considered mutations appearing in less than 5% of samples (see STAR Methods). The model applies for each mutation the IC_50_ values across the different samples that contain it and calculates the relative likelihood of that mutation to contribute to resistance (see STAR Methods and Figure S4C). A total of 17 unique mutations were identified by this approach (see Figure S4D and Data S5). In addition, to account for mutations that are not represented in our datasets, we used the structure of the TMR-bound Env (5U7O) to identify Env positions with side chains that extend into the CD4-binding pocket (Table S2). We then examined the frequency of all residues at these positions in HIV-1 clade B viruses, represented by a panel of 2,535 Envs from fostemsavir-untreated individuals (see alignment in Data S6). Variants that appeared in at least 0.5% of this panel were selected. We chose this cutoff to focus on Env substitutions supported by multiple observations, which reduces the influence of sampling noise and sequencing artifacts. Such a threshold provided confidence in fitness of the viruses that contain the mutations among diverse HIV-1 isolates. A total of 17 such unique mutations were identified. Finally, we identified 11 unique mutations noted in previous studies to increase resistance to TMR or its structural analogs^32,34,35,38,46,47^ (Table S3). The four approaches yielded a total of 59 unique mutations at 21 Env positions that were suspected of increasing Env resistance to TMR (Figure 2E).

### Phenotypic analysis of mutations suspected of increasing HIV-1 resistance to TMR

**Figure 3.**
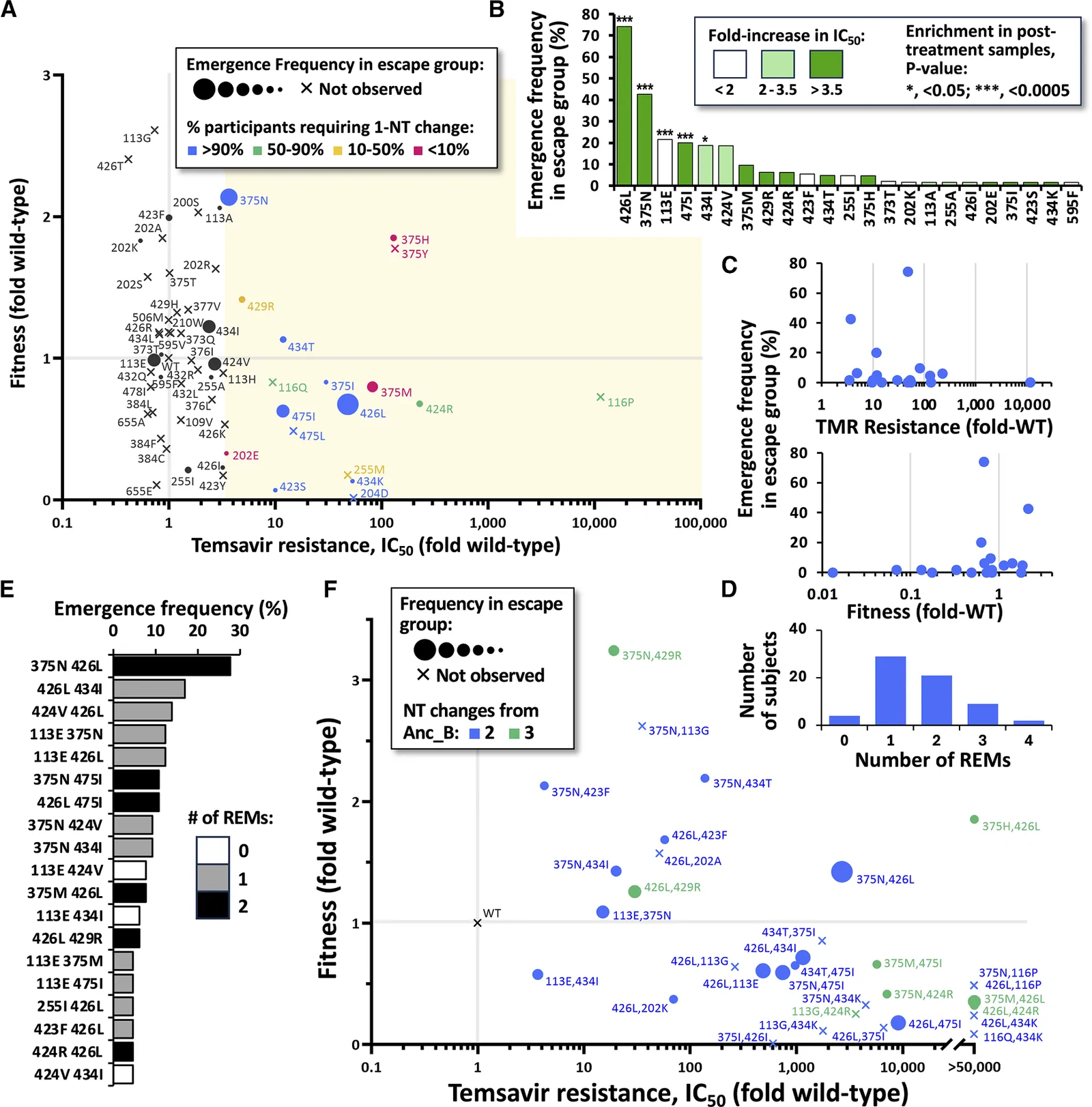
Differential emergence frequencies of mutations that confer resistance to TMR among treated individuals (A) The 59 mutations suspected of increasing resistance to TMR were introduced in the AD8 Env and tested in a pseudovirus system for fitness (infectivity normalized to particle content) and TMR resistance. Datapoint size reflects emergence frequency in the escape group. Color indicates the percent of BRIGHTE subjects that required one nucleotide (NT) change to acquire the mutation. (B) Emergence frequency of the mutations in the escape group. Significance of mutation enrichment in the post-treatment samples, as determined in a permutation test, is indicated. (C) Relationship between frequency of the 18 mutations that increase resistance by more than 3.5-fold (designated REMs) and their effects on TMR resistance or fitness. (D) Number of REMs that emerged in each of the 65 escape group subjects. (E) Emergence frequency of mutation combinations in the escape group. (F) Fitness versus TMR resistance for two-mutation combinations. Datapoint size reflects frequency in the escape group; color indicates the number of NT changes required to acquire the mutation from the clade B ancestral sequence.

To determine the effects of the 59 mutations on Env fitness and TMR resistance, we introduced them individually into the Env protein of HIV-1 strain AD8. This well-characterized clade B strain, which is strictly CCR5-tropic,^48^ occupies a closed conformation typical of Tier-2-like primary HIV-1 isolates.^49,50^ Pseudoviruses that contain the mutant Envs and express the luciferase gene were used to infect Cf2Th cells engineered to express CD4 and CCR5. Luciferase activity was then normalized to virus particle content by reverse transcriptase activity in each sample.^51^ Finally, this value was expressed relative to this ratio measured for the wild-type AD8 Env in the same experiment (Figures S5A and S5B). All 59 mutants were also tested for their resistance to TMR. In addition, for each mutation, we examined the emergence frequency after treatment in the escape group, as well as the proportion of the 65 participants that required a single-nucleotide change to acquire the mutation. As shown in Figure 3A, most suspected mutations did not alter resistance to TMR, while several increased it considerably (e.g., L116P). Interestingly, some mutations were significantly enriched in the escape group, most notably 426L and 375N, which appeared in 74% and 38% of these individuals, respectively (Figure 3B). To better understand the basis for the differential emergence frequencies of mutations after treatment, and based on the observed variability in the IC_50_ values, we focused on the subgroup of 18 mutations that increased the IC_50_ by 3.5-fold or more (see shaded region in Figure 3A). We designate this subgroup REMs. For some REMs (e.g., 375H, 375M, and 375Y), their low frequency in the escape group could be explained by the number of nucleotide changes required (see color of data points in Figure 3A). However, several poorly sampled REMs (e.g., 375I) only required a single-nucleotide substitution. REM emergence frequencies were not associated with their effects on resistance to TMR (Figure 3C). By contrast, a threshold effect was observed for fitness, whereby low-fitness REMs (e.g., 434K, 255M, and 204D) were poorly sampled, whereas REMs with favorable fitness profiles and nucleotide substitution requirements showed variable frequencies of emergence.

In most individuals, REMs appeared at more than one Env position (Figure 3D). We thus examined the fitness-resistance profiles of the two-site mutation combinations that appeared frequently after treatment, as well as combinations that appeared less frequently or not at all (Figures 3E and 3F). The most frequent combination in the escape group, 375N/426L, which requires one nucleotide substitution in each codon, exhibited a favorable fitness-resistance profile relative to other two-substitution combinations. Nevertheless, it did not exhibit a unique synergistic advantage in fitness or TMR resistance that could explain the observed high prevalence of this combination or of the individual changes (Figures S5C, S5D, and S5E).

In the above tests, we compared the emergence frequencies of REMs with their fitness and TMR resistance levels measured using a single HIV-1 isolate (strain AD8). Nevertheless, it could be argued that some poorly sampled REMs, such as 116P, may only increase Env resistance in the context of a limited number of strains (e.g., AD8). Conversely, the 113E and 434I mutations, which appeared frequently after treatment but did not increase AD8 Env resistance significantly, could exhibit limited effects in AD8 relative to other strains. To address this concern, we introduced the 116P, 113E, and 434I mutations into the Envs of two clade B-transmitted/founder strains, 700010040.C9.4520 and WEAUd15.410.5017.^50^ For the 113E mutation, which emerged frequently in BRIGHTE, we also tested the Envs of strains QH_692 and JRFL. For all three mutations, the effects on fitness and resistance were qualitatively similar to those observed for AD8 (Figures S6A and S6B). While these tests included only 2–4 variants, they suggested that the effects of the mutations observed in the Env of strain AD8 can be generalized to other clade B isolates.

To better understand the basis for the frequent emergence of the D113E and M434I mutations on treatment despite limited effects on TMR resistance, we examined their effects alone and in combination with the 375N and 426L changes. As shown in Figure S6C, both mutations enhanced the effects of the 375N, 426L, and 375N/426L variants considerably. Such enhancement may explain the finding that 375N/426L/113E was the most frequent triple mutation combination that appeared on treatment. These results support the notion that 113E and 434I play an enhancer role. Alone they do not dramatically increase resistance to TMR; instead, they synergize with REMs to enhance their effects. Indeed, 113E and M434I appeared most frequently with or after REMS at positions 375 and 426 (in 14 and 13 participants, respectively) rather than before appearance of these REMS (in 2 participants each).

In summary, we identified 18 mutations that increase Env resistance to TMR. Some REMs emerged after treatment at significantly higher frequencies than others. This preference could not be fully explained by the number of nucleotide changes required or by the level of resistance to TMR they impart, and only partially by their effects on Env fitness.

### The emergence frequency of REMs in the escape group corresponds with their spontaneous emergence rate in the population

**Figure 4.**
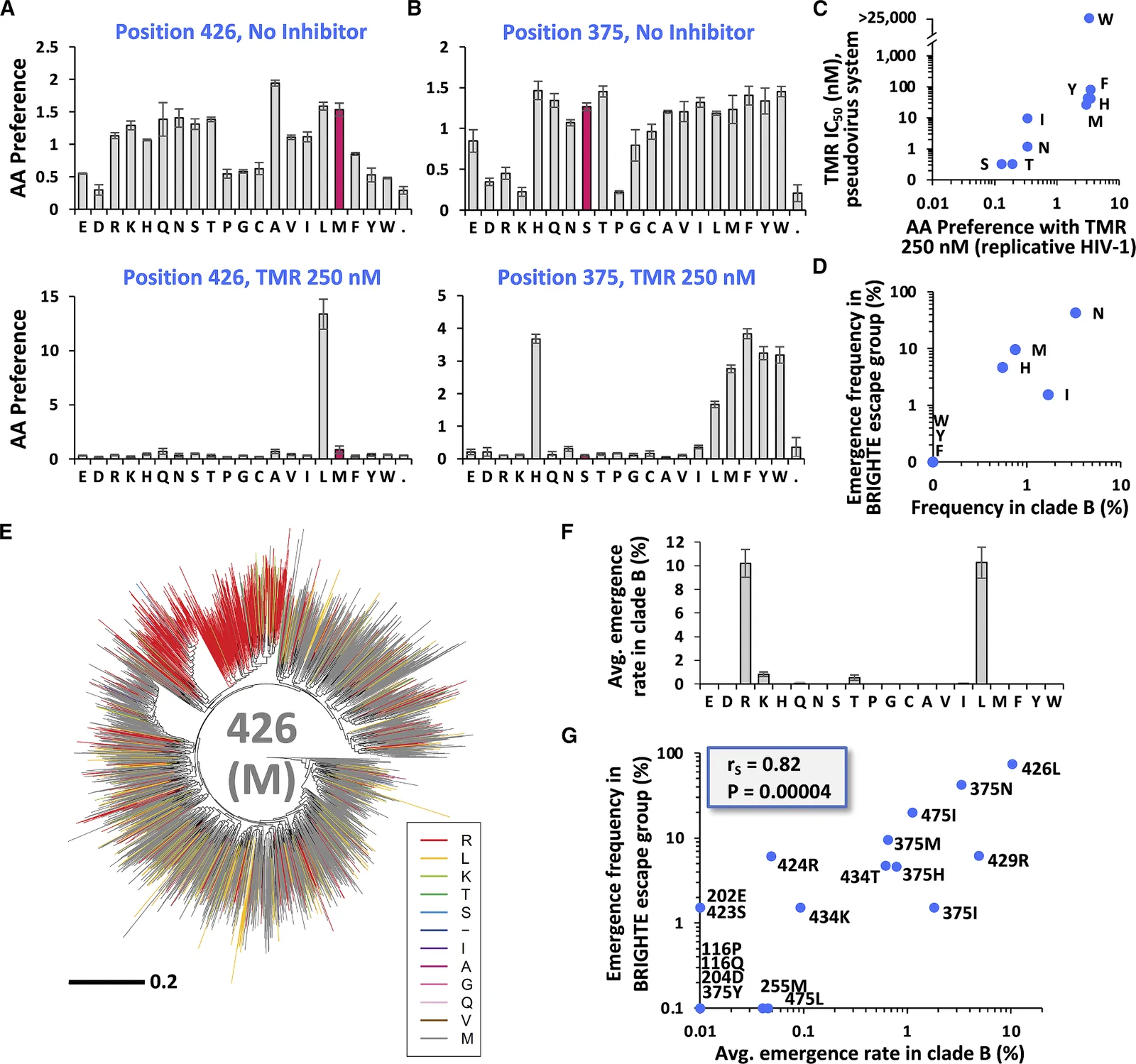
The frequency of REM emergence in the escape group corresponds with their spontaneous emergence frequency in the population (A and B) Saturation mutagenesis to determine the effects of all amino acid changes at positions 426 and 375 on HIV-1 fitness (“No inhibitor”) and resistance to TMR (250 nM). The wild-type form is shown in maroon. (C) Relationship between the preference for amino acids at position 375 in the presence of TMR and their IC_50_ values measured using the pseudovirus system. (D) Relationship between the emergence frequency of amino acids at position 375 in the escape group and their frequency in a panel of 2,535 clade B Envs from fostemsavir-untreated individuals. (E and F) Example of the emergence rate of mutations at position 426 in HIV-1 clade B. The tree was constructed using amino acid sequences of the 2,535 Envs. Branches are colored by the amino acid in each taxon at position 426. The tree was partitioned into subgroups, which were excluded if the dominant form at that position differed from the clade B consensus. For all remaining subgroups, the number of new substitution events at position 426 to each amino acid was calculated, and these values were averaged. Error bars, standard errors of the mean. (G) Relationship between emergence frequency of the 18 REMs in clade B and their emergence frequency in the BRIGHTE escape group.

The 426L mutation, which appeared in 74% of subjects in the escape group, increased TMR resistance by 48-fold, while four other suspected REMs at this position (Ile, Thr, Lys, and Arg) did not increase resistance significantly (Figure 3A). To assess the comprehensiveness of our screening approach, we examined if additional variants at this position may also increase resistance to TMR. To this end, we performed saturation mutagenesis to test the effects of all possible mutations at position 426 on Env fitness and resistance to TMR.^52^ Replication-competent libraries of HIV-1_AD8_ that contain a degenerate codon at this position were used to infect the T cell line A3R5.7 in the absence or presence of TMR (250 nM). The frequency of each form in the infected cells was compared with the frequency in the virus library used for infection (see protocol in STAR Methods). While several residues at this position showed fitness levels similar to the wild-type Met, remarkably, only Leu was able to infect the cells in the presence of this concentration of TMR (Figure 4A).

We also tested the fitness and TMR resistance profiles of all residues at position 375 (Figure 4B). REMs 375H, 375I, 375M, 375N, and 375Y, which showed considerable increases in resistance using the pseudovirus system (Figure 3A), also showed high resistance in these experiments (see correlation in Figure 4C). Interestingly, in addition, the 375W and 375F variants showed high resistance to TMR. These forms were not identified by our four approaches because they did not appear in the sequence-IC_50_ datasets or in the panel of 2,535 clade B isolates from fostemsavir-untreated individuals. Indeed, the emergence frequency of mutations at position 375 in BRIGHTE participants correlated well with their proportion in the fostemsavir-untreated clade B population (Figure 4D). To better estimate the emergence frequency of the REMs in fostemsavir-untreated individuals, we analyzed a panel of 2,535 clade B Env sequences. A phylogenetic tree was constructed and divided into 15 subgroups representing independent evolutionary lineages. Each Env position was analyzed separately by assigning each sample in the tree the amino acid present at that site (see example for position 426 in Figure 4E). Subgroups in which the non-ancestral residue predominated were excluded to avoid counting mutations that had already become established within a lineage. For each REM, the average number of independent emergence events (from the inferred ancestral sequence) was then calculated across the remaining subgroups (Figure 4F). A strong correlation was observed between the average rate of spontaneous REM emergence in clade B and their emergence in the escape group (see Figure 4G and amino acid frequency distributions in Figure S7). These findings suggested that the same selection pressures exerted in fostemsavir-untreated individuals may determine the likelihood of the REMs to emerge on treatment.

### Immune pressures restrict the escape paths of HIV-1 from TMR

**Figure 5.**
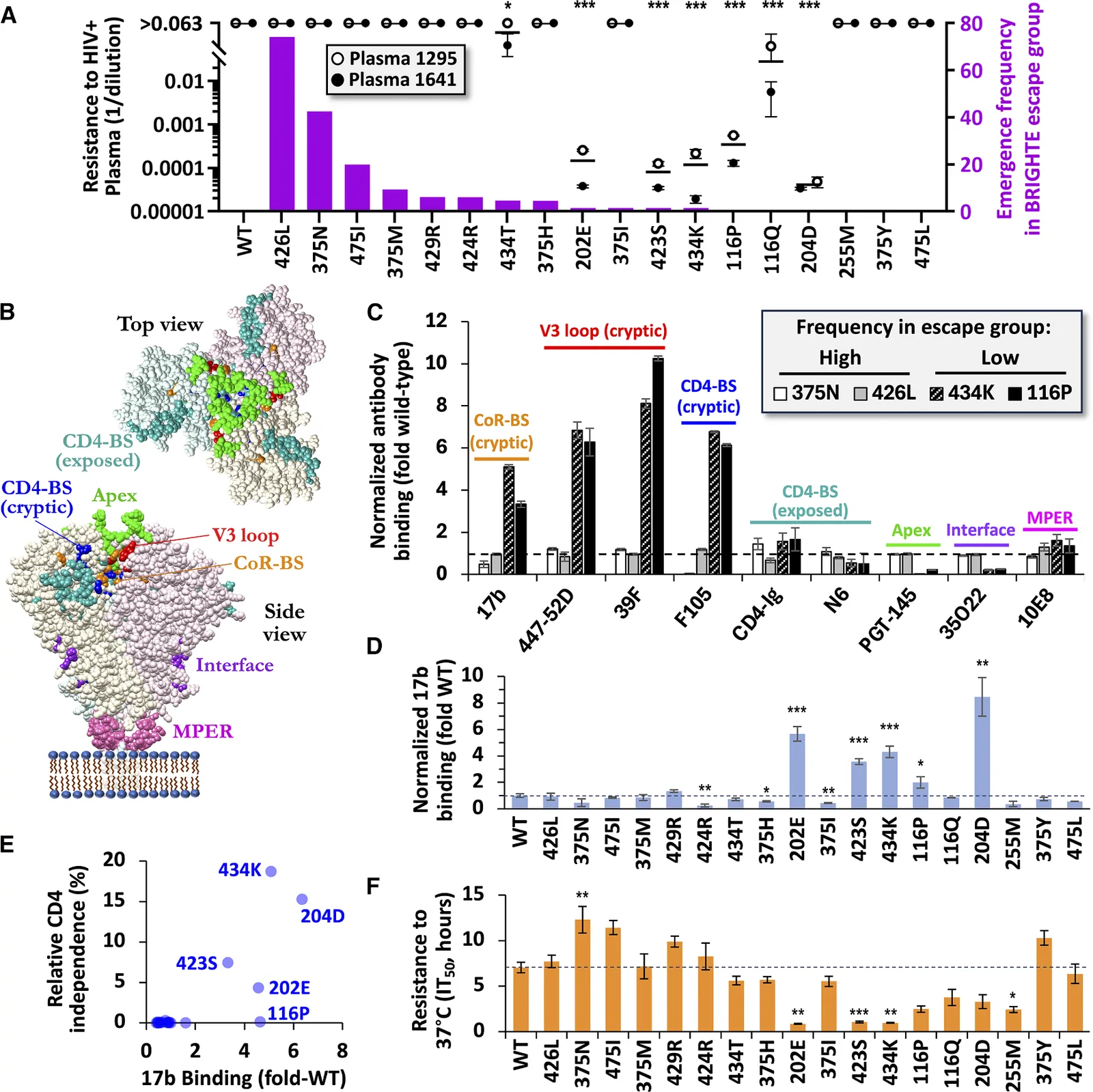
Infrequent REMs in the BRIGHTE escape group exhibit an open Env conformation sensitive to non-neutralizing antibodies (A) Sensitivity of the 18 REMs to plasma from two HIV-infected individuals. Differences from the WT Env were assessed using a mixed effects model: *p < 0.05, ***p < 0.0005. Emergence rates in the escape group are shown as magenta bars. (B) Cryo-EM structure of the Env trimer ectodomain in the CD4-unliganded state (PDB IDs 6U59 and 6UJV). Residues associated with binding of the tested antibodies are colored. (C) Envs containing the indicated REMs were expressed on HOS cells, and binding of monoclonal antibodies was measured by cell-based ELISA. Values were normalized for cell surface expression using antibody 2G12 and are expressed relative to the wild-type AD8 Env. (D) Binding efficiency of the 18 REMs to antibody 17b that targets the cryptic gp120 CoR-BS. *p* values in an unpaired *t* test: *p < 0.05, **p < 0.005, ***p < 0.0005. (E) Relationship between binding of the REMs to 17b and their infection of CD4-negative cells, expressed as a percent of their infection of CD4-positive cells. (F) Resistance to incubation at 37°C, shown as time to 50% loss of infectivity. *p* values, unpaired *t* test relative to the wild-type Env. *p < 0.05, **p < 0.005, ***p < 0.0005.

Selection pressures on replicative fitness and inhibitor resistance are primary factors that guide the evolution of virus proteins in treated individuals. Interestingly, the relationships shown in Figure 3C only suggested a threshold effect for fitness and none for the level of resistance imparted by the REMs. We thus sought to identify additional factors that may contribute to the observed preference for some REMs to emerge (on treatment and in the clade B population). In most HIV-infected individuals, antibodies are elicited against conserved epitopes that overlap the coreceptor-binding site (CoR-BS), CD4-binding site (CD4-BS), and inner domain of gp120.^53,54^ These epitopes are not exposed on the native state of Env in most primary isolates, and are thus designated non-neutralizing. The non-neutralizing antibody response was suggested to reduce the risk of infection in vaccine trials^55,56^ and alter the evolutionary path of HIV during early stages of infection.^57–59^ In both cases, the attributed mechanism was based on Fc-mediated effector functions. To examine the potential effect of such antibodies on mutation emergence during treatment, we measured the sensitivity of the REMs to plasma samples from two HIV-positive individuals that do not reduce infectivity of Env AD8 at the lowest dilution tested (1:160). As shown in Figure 5A, several REMs that did not emerge on treatment were neutralized by the plasma (e.g., 116P and 434K), whereas REMs that were sampled more frequently were resistant.

To determine the epitope specificity of the antibodies in the plasma that differentially neutralize these mutants, we performed a preliminary experiment using the frequently sampled (plasma-resistant) 426L and 375N mutants and the poorly sampled (plasma-sensitive) 116P and 434K mutants. Plasmids that encode the Envs were used to transfect human osteosarcoma (HOS) cells, which express on their surface fully cleaved Env trimers in their native closed form.^60^ Monoclonal antibodies that target different Env epitopes (Figure 5B) were added to the cells, and their binding efficiency was detected by cell-based ELISA.^61^ As shown in Figure 5C, the 116P and 434K changes enhanced binding of antibodies against otherwise-cryptic epitopes that overlap the CoR-BS, V3 loop, and CD4-BS. In addition, these changes reduced binding of antibody PGT145 that targets a quaternary epitope at the apex of the trimer.^62^ These features are consistent with a CD4-bound-like conformation of Env.^63,64^ We extended the analysis to all 18 REMs and observed that, in addition to 116P and 434K, mutations 423S, 202E, and 204D, which appeared infrequently in the escape group and were sensitive to non-neutralizing plasma (Figure 5A), also increased binding of the CoR-BS antibody 17b (Figure 5D and the relative expression levels of the Envs in Figure S8A). Given the CD4-bound-like conformation of these REMs, we also tested their ability to infect CD4-negative cells.^49^ Indeed, a strong relationship was observed between this variable and the level of 17b binding (Figure 5E; Figure S8B). Interestingly, only the 116P mutant showed high binding of 17b but no CD4-independent infection, suggesting two potential structural-functional outcomes for these changes.

We also examined the effects of the 18 REMs on Env stability. The Envs of diverse HIV-1 isolates can exhibit different levels of conformational stability^49,61^ and sensitivities to inactivation at physiological temperature.^65,66^ Nevertheless, a relationship between stability of Env variants and their likelihood to appear in HIV-infected individuals has not been established. To this end, we incubated viruses containing the 18 REMs at 37°C and measured the changes in residual infectivity over time. Consistent with previous results for the related ADA strain,^66^ the half-life of the wild-type AD8 Env was approximately 7 h (Figure 5F). Interestingly, the infrequently sampled REMs that were sensitive to non-neutralizing plasma and enhanced exposure of the CoR-BS also demonstrated low functional stability at 37°C (see correlations in Figure S9A).

Taken together, these results suggest that REMs 116P, 434K, 204D, 202E, and 423S increase sensitivity to non-neutralizing plasma by inducing an open (CD4-bound-like) form of Env that exposes cryptic epitopes targeted by non-neutralizing antibodies. These mutations also reduce Env stability and functional fitness. Their enhanced resistance to TMR can be explained by a change in the CD4-binding pocket to a CD4-bound-like conformation that is not conducive with binding of TMR (note location of the SM^3^ sites in Figures S9B, S9C, and S9D). However, their resistance is associated with a cost—elimination by the non-neutralizing antibody response.

### A combined model to predict the escape path of HIV-1 in treated individuals

**Figure 6.**
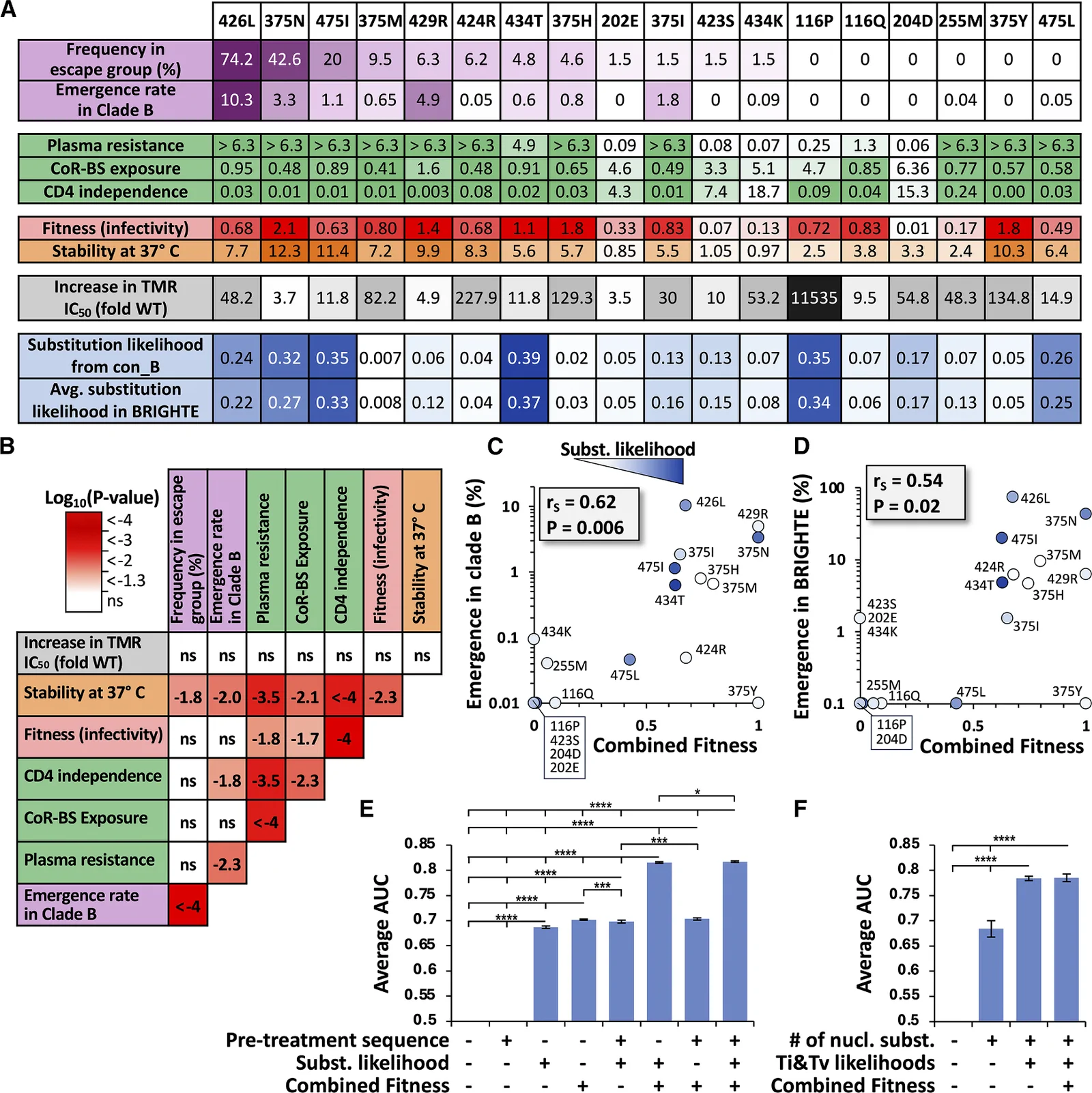
Virus and host factors that guide emergence of REMs (A) Summary of all features measured for the 18 REMs. Substitution likelihoods from the clade B consensus (con_B) trinucleotide sequence describe the number of nucleotide changes required and the transition/transversion rate for each change. This likelihood was also calculated using the pre-treatment nucleotide sequences of the 65 subjects of the escape group. (B) *p* values for a Spearman correlation test that compares the values shown in (A). ns, not significant. (C and D) The combined fitness metric for each REM was calculated as the product of their effects on fitness, resistance to plasma, and stability at 37°C. This value was compared with REM emergence frequencies in clade B or in the escape group. (E) Simulations of REM emergence were performed with different combinations of the indicated variables. The fraction of success events (mutation acquisition) in the 1,000 iterations was compared with REM frequency in the 65 subjects (see algorithm flowchart in Figure S10C). Data for all 18 REMs were compiled to calculate the AUC. Error bars, SDs for 10 simulations. *p* values in an unpaired *t* test: *p < 0.05, ***p < 0.0005, ****p < 0.0005. (F) Contribution of the number and type of nucleotide substitutions to predict emergence of the five REMs at position 375 (H, I, M, N, and Y). *p* values, unpaired *t* test. ****p < 0.00005.

We examined the relationships between the above-described features of the 18 REMs, including their emergence frequencies during treatment and in the clade B population (see values in Figure 6A and *p* values for the Spearman correlation tests in Figure 6B). We divide these features into four groups: (1) functional fitness, measured by the fusion competence of the Env; (2) functional stability, measured by resistance to inactivation at 37°C; (3) immune fitness, captured by exposure of the CoR-BS and sensitivity to the non-neutralizing plasma and indirectly, by the requirement for CD4 to infect cells; and (4) therapeutic fitness, measured by the *in vitro* resistance to TMR. Strong correlations were observed between the variables that capture functional fitness, stability, and immune fitness. However, none of them alone could fully explain the emergence frequency of the 18 REMs in the escape group, other than a modest correlation with Env stability at 37°C. As such, we decided to generate a combined fitness variable that describes the functional fitness, stability, and immune resistance of each REM. It was calculated as the product of their relative infectivity, plasma resistance, and stability at 37°C (expressed as a fraction of the values measured for the wild-type Env). As shown in Figures 6C and 6D, the combined fitness of the REMs correlated well with their emergence rates in the BRIGHTE participants and in clade B. Yet some REMs, such as 375Y, showed high combined fitness but did not emerge on treatment.

Based on these findings, we also examined the likelihood of the mutations to appear, as determined by (1) the pre-treatment nucleotide sequence of the participants, (2) the number of nucleotide changes needed to acquire each REM, and (3) the type of nucleotide changes required, based on the transition and transversion rates determined by Martinez Del Rio et al.^15^ (Figure S10A). An algorithm that accounts for these variables was generated, in which mutation appearance was modeled as a series of stochastic events with mutation-specific probabilities (see flowchart in Figure S10B). As shown in Figure 6A (bottom rows), similar REM appearance likelihoods were calculated using the clade B consensus and BRIGHTE pre-treatment sequences as the starting state. We examined if these likelihoods could explain the outliers in Figures 6C and 6D (see colors of datapoints). As expected, several REMs with high fitness but low emergence frequencies (most notably 375Y, 424R, and 375I) exhibited low substitution likelihoods.

We extended our analyses to account for the two steps associated with emergence of each REM: appearance and persistence (see flow chart in Figure S10C). These two components were combined with the pre-treatment trinucleotide sequence at each site to predict the frequencies of the 18 REMs in the escape group. The likelihood for appearance was calculated by the number and type of the nucleotide substitutions. The likelihood for REM persistence was captured by a combined fitness metric that incorporates the functional fitness, stability, and immune fitness. As shown in Figure 6E, the model that integrated all variables demonstrated good predictive performance (AUC = 0.82) and was robust across multiple cross-validation folds, as indicated by the error bars. The combined fitness variable and the nucleotide substitution likelihoods contributed to the prediction significantly. Consistent with the data in Figure 6A, the use of the participants’ pre-treatment sequences did not improve performance relative to the clade B consensus sequence.

Finally, we explored the contribution of the nucleotide substitution likelihoods to the observed profile of mutations in BRIGHTE. To this end, we examined separately the effects of the number of changes required and the probability for each transition or transversion.^15^ For these tests, we focused on position 375, which contains the largest number of REMs. Interestingly, both the number of changes and the expected rate of each substitution contributed to predictions of REM emergence (Figure 6F). For example, position 375 was occupied in most subjects by Ser with the trinucleotide sequence AGT (or less frequently by AGC). Substitutions to Asn (AAT/AAC) or Ile (ATT/ATC) constitute single-nucleotide changes (from G to A or G to T, respectively). However, the G to A transition is more common than the G to T transversion, likely explaining the higher frequency of Asn in the escape group and population relative to Ile despite favorable fitness profiles in both. Indeed, given the similar immune and functional fitness levels for the five REMs at position 375 (Figure 6A), addition of the selection pressures did not further improve prediction of the on-treatment mutational outcomes (Figure 6F). We note that these calculations are not intended to capture the quantitative effects of the variables on appearance or persistence of the REMs. Nevertheless, they clearly demonstrate that the mutational path is explained well by the properties of Env measured *in vitro* and modeled *in silico*.

## DISCUSSION

### The forces that guide the path of HIV-1 escape from a therapeutic *in vivo*

Through a comprehensive screen, we identified and then tested 59 mutations suspected of increasing HIV-1 resistance to TMR, of which 18 REMs were found to be significantly impactful. Analysis of the utilization of these paths in individuals treated with fostemsavir revealed that some were highly preferred, whereas others, despite favorable fitness and resistance profiles, were sampled infrequently or not at all. Given that the emergence frequency of the REMs in BRIGHTE corresponded well with their spontaneous appearance rates in fostemsavir-untreated individuals, we hypothesized that additional selection pressures may restrict some paths of escape. Indeed, several REMs poorly represented *in vivo* exhibited “open” forms of Env; their mechanism of resistance is based on induction of a CD4-bound-like conformation that is not conducive with binding of TMR. However, the penalty associated with this path is exposure of otherwise-cryptic epitopes that overlap the CoR-BS and V3 loop. Such forms are readily eliminated by non-neutralizing antibodies that are commonly elicited in HIV-infected individuals.^53,54^ For other REMs, most notably at position 375, their poor representation in the escape group was explained by the low likelihood of the substitutions to occur, due to the number or type of nucleotide changes required. Thus, we quantitatively capture the *in vivo* factors that impact the two phases of virus escape from a therapeutic: (1) *appearance* of the mutation, as determined by the number and type of nucleotide substitutions, and (2) *persistence* of the new variant, as determined by the effects on functional fitness, immune fitness, and stability of the protein. Interestingly, a very high level of resistance imparted by some REMs (e.g., 116P) was not able to overcome low levels of functional and immune fitness. The ability to explain the path of resistance by effects of the individual mutations strongly suggests a limited contribution of adaptive changes to escape of the virus. Indeed, REMs with poor functional or immune fitness were rarely observed *in vivo*. Instead, for up to 5 years of follow-up, the mutations with the most favorable profiles emerged. These findings are consistent with the catastrophic effect of the most readily available mechanism of resistance—transition to a CD4-bound like conformation.

### Role of the non-neutralizing antibody response in restricting escape paths from a therapeutic

An unexpected finding of this study is the effect of non-neutralizing antibodies on the range of mutations that can mediate escape. The neutralizing response against HIV-1 Env is usually swarm specific and exhibits limited cross-neutralization with other strains. By contrast, antibodies that target otherwise-cryptic epitopes in the V3 loop and CoR-BS are commonly elicited across different hosts.^67,68^ We designate such antibodies non-neutralizing, as they do not inhibit infection by closed Tier-2-like strains that predominate in HIV-infected individuals. Our results suggest that it indeed constitutes a deterministic pressure that guides the evolutionary path of HIV-1, similar to the requirement for functional fitness. Such pressure is consistent with the inability to isolate CD4-independent primary strains from HIV-infected individuals, despite the clear advantage associated with overriding the need for the CD4 receptor.^69^ The five REMs that were poorly sampled in the escape group (116P, 434K, 202E, 204D, and 423S) induced an open form of Env that exposes the CoR-BS, which likely increased their sensitivity to non-neutralizing antibodies against this epitope in the HIV-positive plasma we used. Interestingly, three of these sites (positions 116, 434, and 204) form a clasp-like structure that appears to stabilize Env in the native untriggered state (Figures S9B, S9C, and S9D). Similar interactions across the gp120 and gp41 subunits have been noted to maintain trimer stability, allowing Env to retain the high potential energy required to drive the fusion process.^70,71^ Weakening of these interactions results in transitions to lower-energy metastable CD4-bound-like forms, which, in the absence of a coreceptor, undergo spontaneous and irreversible conformational changes into non-functional states.^72^ Indeed, here we observe strong relationships between the functional fitness of the variants, their level of “openness,” and their stability at 37°C. Given the large number of such Env-stabilizing interactions, it is plausible that mutations at other sites, outside the CD4-binding pocket, may also increase resistance to TMR. While these mutations may emerge during *in vitro* selection experiments, they appear to be restricted during *in vivo* escape from this therapeutic.

### Effects of Env structural context on availability of escape paths

Env exhibits tremendous diversity in amino acid sequence within and between hosts.^17,18^ Divergent profiles of antigenicity and networks of allosteric interactions result in distinct profiles of sensitivity to therapeutics. In addition, the fitness profile of Env positions may vary between strains, including those derived from the same HIV-1 clade.^52^ As such, an REM could exhibit high fitness in the context of one strain but low fitness in another, restricting viability of that escape path. Context specificity can limit the ability to infer, for example, from our tests with the AD8 Env to other HIV-1 strains. To better understand the high frequency of two mutations that do not increase TMR resistance in our assays (113E and 434I), and the absence of REM 116P that increases resistance significantly, we tested their effects on additional isolates. Qualitatively similar effects were observed on fitness and TMR resistance of these strains, suggesting that, at least for the CD4-binding pocket, the effects measured for Env AD8 are generalizable. This notion was also supported by the observation that the frequency of most REMs in the escape group was explained well by their appearance and persistence likelihoods based on the *in vitro* data measured for AD8 Env. An exception to this rule is REM 475L, which exhibited a relatively high likelihood for appearance and persistence but was poorly represented in the BRIGHTE escape group. Consistent with this finding, 475L only appeared in one of the 2,535 clade B isolates from fostemsavir-untreated individuals. Comprehensive characterization of the effects of this mutation on Env functional and immune fitness in diverse strains, as well as the potential for other selection forces such as the cytotoxic T lymphocyte response,^73,74^ may provide greater insight into the basis for the low emergence rate of this mutation.

Based on prior studies of CD4-mimetic compounds,^49,61,70^ we expected to identify mutations outside the pocket that contribute to TMR resistance. However, all variants enriched in the escape group as well as those that increased the *in vitro* resistance to TMR localized to the pocket vicinity. Mutations outside this domain could plausibly exert long-range effects by inducing an open form of the Env trimer; however, such variants are not viable *in vivo* as they are cleared by the non-neutralizing antibody response. While previous studies identified REMs in gp41 (see Table S3; Figure 2E), the mutations emerged *in vitro*, in a background of the neutralization-sensitive (Tier 1B) lab-adapted strain LAI.^35,47^ This Env occupies a partially open conformation of the trimer,^75^ which was likely further destabilized by the gp41 mutations. Indeed, when tested in the background of the Tier-2 Env AD8, they did not increase TMR resistance. Thus, we cannot exclude the existence of mutations that can modulate pocket structure and reduce engagement of TMR while maintaining Env in the closed form, but such variants were not identified across the complementary approaches used here.

### Personalization of antiviral treatments based on virus genotype

Several sites located in the CD4-binding pocket show considerable within-clade variation relative to that expected for the critical function of this domain (Table S2). Most prominent are positions 426 and 375, which also harbor the two most frequent REMs. Both sites exhibit broad fitness profiles, as measured by saturation mutagenesis (Figures 4A and 4B). For example, the ancestral Met at position 426 is often replaced by Arg and Leu (Figure 4F); both are fit, but only Leu is resistant to TMR. Similarly, several variants at position 375 can impart resistance and exhibit high levels of functional and immune fitness (Figure 6A); however, they are restricted by a low likelihood for appearance, due to the number (His, Met, and Tyr) or type (Ile) of nucleotide changes required. The frequencies of 426L and 375N in HIV-1 clade B increased at early stages of the AIDS pandemic and seem to have reached steady states, likely reflecting the lack of an advantage relative to the ancestral forms (Figure S11). Thus, the prevalence of variants that enhance resistance to TMR, and potentially other inhibitors against the CD4-binding pocket,^76,77^ are not low but are also not increasing. Indeed, 24 of the 132 subjects that were sampled before and after treatment showed resistance to TMR prior to initiation of therapy, and 65 developed resistance on treatment (Figure 1F). Of all 358 BRIGHTE participants with pre-treatment samples, 52 had pre-existing resistance to TMR (14.5%). The high emergence frequencies of 426L and 375N reflect their favorable immune and functional fitness profiles and suggest that future inhibitors targeting the CD4-binding pocket should be designed to maintain efficacy against variation at these positions.

A long-standing goal in the field of infectious disease management is development of tools to personalize antiviral therapeutics by rapidly estimating the properties of the infecting virus swarm before treatment.^78–81^ The non-negligible frequency of TMR-resistant strains in the population and the high rate of resistance that emerges on treatment suggest a need for such tools. Nevertheless, the diversity and complexity of Env can challenge our ability to predict resistance from sequence data.^82^ As we show here, HIV-1 sensitivity to TMR can be readily inferred from the amino acid sequence of Env, with AUC values exceeding 0.95. Given the limited effects of Env context on these predictions, our algorithms can be applied to strains from diverse HIV-1 clades. An example of the relationship between the predicted and measured IC_50_ values using the GB regressor model is shown in Figure 2D. For predicting resistance above 50 nM TMR, the false-positive rate was 3% and the false-negative rate was 7.1%. Notably, the linearity of the relationship between the probabilities for resistance and the measured IC_50_ values allows us also to consider the estimated level of resistance during clinical decision-making. Such performance is high relative to previous analyses of epitopes targeted by BNAbs,^82–84^ which may be attributed to the relatively low complexity of the TMR epitope or the restricted range of mutations that can impart resistance and persist in the host.

The sequence data derived from the HIV Envelope NGS Sequencing Assay and applied in this study does not capture the full diversity of plasma virus variants in each participant. Indeed, genotypic profiling assays used clinically are generally limited to detection of mutations with frequencies greater than 20% of all sequences.^85,86^ We expect that sequencing of plasma virus at greater depth will provide the accuracy required to predict initial clinical responses as well as short-term emergence of resistant forms from low-frequency circulating variants. Processing of clinical samples by deep sequencing followed by sequence-based estimation of resistance, as described here, can be achieved with limited expense in short time frames relative to phenotypic *in vitro* assays. The approach has the potential to improve both the time-to-treatment initiation and the efficacy of personalized TMR treatments. Furthermore, if resistant forms are not detected in the pre-treatment sequences, the data can be used to quantitatively describe the likelihood for emergence of each resistance mutation, based on the nucleotide substitution requirements and the estimated effects on functional and immune fitness of the virus.

We propose that a modular approach to isolate and analyze the factors that contribute to a variant’s likelihood to persist can be applied to other HIV-1 proteins and treatments. Indeed, the overall fitness of any viral protein can be divided into distinct, measurable axes of fitness. For example, for reverse transcriptase (RT) inhibitors, viral titers measured for RT mutants *in vitro* describe their ability to infect, but will not capture mutation effects on fidelity of the enzyme or on template switching, which are deleterious for the virus. Similarly, for integrase inhibitors, *in vitro* titers can capture the ability of the mutants to mediate genomic integration, but not effects on integration site distribution or proviral transcriptional competence. Such effects are measurable properties that can be combined with the overall measured replication capacity of the virus and drug resistance to determine the likelihood for persistence of the new variant.

### Limitations of the study

A limitation of this study is that the sequencing approach utilized does not fully capture low-frequency Env variants. Deeper sequencing may further improve prediction of the mutational profiles that emerge during treatment and early treatment responses. Furthermore, sequence data for other HIV-1 genes were not available. While a linkage between mutations in Env and resistance to different antiretroviral therapy classes has been shown,^87^ mutations in non-Env proteins that increase resistance to Env-targeting agents have not been described to our knowledge. In our study, resistance-associated Env mutations were identified across 65 independently treated patients. In addition, our functional *in vitro* assays demonstrate that the REMs we identified independently confer reduced TMR sensitivity, supporting their role as direct drivers of resistance rather than incidental passengers.

## RESOURCE AVAILABILITY

### Lead contact

Further information and requests for resources and reagents should be directed to the lead contact, Hillel Haim (hillel-haim@uiowa.edu).

### Materials availability

Plasmids that express all described Env mutants and reagents used for the deep mutational scanning experiments are available upon request from the lead contact, Hillel Haim (hillel-haim@uiowa.edu), under a material transfer agreement with the University of Iowa.

### Data and code availability

- Data: Data sequence of the pNL-AD8 plasmid is deposited in GenBank: PV345784. All plasmids used in this study are listed in the key resources table. All data reported in this manuscript will be shared by the lead contact upon request.
- Code: the code to analyze DMS data (EZ-DMS) can be found at https://github.com/haimlab/EZDMS. The code to calculate the frequency of independent emergence events of the REMs can be found at https://github.com/haimlab/Independent_mutation_Tree. The code to simulate emergence of the REMs can be found at https://github.com/haimlab/REM-Emergence-Modeling.
- Other items: any additional information required to reanalyze the data reported in this paper is available from the lead contact (hillel-haim@uiowa.edu).

## STAR★METHODS

### EXPERIMENTAL MODEL AND STUDY PARTICIPANT DETAILS

#### Cell lines

Human embryonic kidney (HEK) 293 T and human osteosarcoma (HOS) cells were obtained from the American Type Culture Collection (ATCC) and were maintained in DMEM supplemented with 1% penicillin/streptomycin and 10% fetal calf serum (designated below PS/FCS) in a 37°C 5% CO_2_ incubator. Cf2Th CCR5+ and Cf2Th CD4+CCR5+ cells were kindly provided by Joseph Sodroski and were maintained in DMEM/PS/FCS supplemented with 0.4 mg/mL of G418, and 0.4 mg/mL of G418 and Hygromycin, respectively. The A3R5.7 cell line was obtained from BEI Resources and was maintained in RPMI supplemented with PS/FCS. TZM-bl cells were obtained from BEI resources and maintained in DMEM/PS/FCS. For all cells lines obtained from the ATCC, BEI Resources and Dr. Sodroski, authentication was not independently performed in our laboratory. All cell lines were tested and confirmed to be free of mycoplasma contamination.

#### Plasma samples from HIV-1 infected individuals

Two de-identified samples of peripheral blood plasma from HIV-infected adults were used in this study. Samples were obtained after informed consent under clinical protocols approved by the participating institutions’ human use review boards, including those at the University of Washington at Seattle Center for AIDS Research (CFAR) and University of Iowa (IRB numbers 8807313, 200010008, and 2010101730). No demographic information (including age, sex, race, or ethnicity) of the donors are available.

#### Virus sequence data from the BRIGHTE trial

This work involved in part *post hoc* analysis of de-identified HIV-1 sequence data from samples collected for the BRIGHTE clinical trial (NCT02362503). No participant-level demographic information (including age, sex, race and ethnicity) was made available to us.

### METHOD DETAILS

#### Production and testing of pseudoviruses containing the env mutations

All 59 mutations suspected of increasing Env resistance to TMR were introduced by site-directed mutagenesis into a pSVIII vector that expresses the Env of strain AD8 under control of the LTR promoter.^93^ All primers are listed in Data S9. Pseudoviruses that contain the variants were generated by transfection of HEK 293 T cells. Briefly, 9.5 × 10^5^ cells were seeded in each 6-well plate well, and transfected the next day with 0.4 μg of the HIV-1 packaging construct pCMVΔP1ΔenvpA, 1.2 μg of the firefly luciferase-expressing construct pHIvec2.luc, 0.4 μg of pSVIII expressing HIV-1 Env, 0.2 μg of pRev expressing HIV-1 Rev, and 4.2 μL of JetPrime reagent (PolyPlus). The medium was replaced the next day, and virus-containing supernatant was collected 24 h later. Samples were cleared of cell debris by centrifugation at 800 × g and filtered through 0.45 μm pore sized membranes.

As a measure of virus particle content, we quantified the reverse transcriptase activity in the samples using a modified version of a previously published protocol,^51^ where TaqMan chemistry was used in place of SYBR Green. In brief, RT-qPCR reactions (25 μL total volume) were prepared with TaqMan Gene Expression Master Mix (ThermoFisher) and contained 2.5 mU of MS2 Bacteriophage RNA (Roche), 1 μM of MS2 Forward Primer, 1 μM of MS2 Reverse Primer, 200 nM of a custom-designed 5′–6FAM-labeled, 3′-TAMRA-quenched TaqMan probe, 0.5 U RNase Inhibitor (Fermentas, EO0381), and 2 μL of pseudovirus sample. Pseudovirus samples were lysed in 0.125% Triton X-100, 50 mM KCl, 100 mM Tris HCl (pH7.4), 0.4 U/μL RNase Inhibitor, and 20% glycerol. A standard curve was generated using a dilution series of recombinant HIV reverse transcriptase (NIH AIDS Reagent Program, Cat. No. 12583) ranging from 10^4^ to 10^12^ pU/μL prepared in the same lysis buffer. Reactions were run in 0.1 mL MicroAmp plates (Applied Biosystems) on a Quant Studio 3 Real-Time PCR System under the following cycling conditions: 42°C for 20 min, 50°C for 2 min, 95°C for 10 min, followed by 50 cycles of 95°C for 15 s and 60°C for 1 min. Virus infectivity was expressed as the mean luciferase activity measured for each virus stock (in relative light units, see below) divided by the reverse transcriptase activity in that sample. All infectivity and neutralization assays were performed using at least two independently generated virus stocks, with each assay performed in triplicate.

#### Measurements of virus sensitivity to TMR, plasma and heat

To measure sensitivity of the variants to TMR (BMS-626529, MedChemExpress) or plasma from HIV-infected individuals, Cf2Th CD4 +CCR5+ cells were seeded in 96-well opaque white plates at 2 × 10^4^ cells per well and infected the next day. For neutralization assays, pseudovirus samples were pre-incubated with the TMR or plasma for one hour at 37°C. Samples were then added to the target cells and incubated for 3 days in a 37°C 5% CO_2_ incubator. To measure infection, the medium was removed, 35 μL passive lysis buffer (Promega) were added, and samples subjected to three freeze-thaw cycles. To measure luciferase activity, 100 μL of luciferin buffer (15 mM MgSO_4_, 15 mM KPO4 [pH 7.6], 1 mM ATP, and 1 mM dithiothreitol) and 50 μL of 1 mM d-luciferin potassium salt (Syd Labs, MA) were added to each sample. Luminescence was recorded using a Synergy H1 microplate reader (BioTek).

To measure the decay rate of virus infectivity at 37°C, pseudovirus stocks were divided into aliquots (one sample for each time point), and all samples were snap-frozen on dry ice immersed in ethanol and stored at −80°C. At different time points, samples were thawed in a 37°C water bath for 2 min and then further incubated at 37°C for 2–48 h. All samples were subsequently added to the Cf2Th CD4+CCR5+ cells and infectivity measured 3 days later by luciferase activity. All tests of infectivity and neutralization were performed in at least three independent experiments that contain three replicates each. Standard errors of the mean were used to quantify the variability in the data.

#### Cell-based ELISA to measure binding of antibodies to cell-surface env variants

To measure effects of the mutations on Env antigenicity, we expressed the different variants on the surface of human osteosarcoma (HOS) cells and measured binding of monoclonal antibodies by cell-based ELISA, as we described.^7,61,94^ Briefly, HOS cells were seeded in 96-well plates (1.4×10^4^ cells per well) and transfected the next day with plasmids that express Env, Tat, and Rev, using 60, 11, and 6 ng of each plasmid per well, respectively, and 0.12 μL per well of JetPrime reagent. Background antibody binding was quantified using cells transfected with a pSVIII construct containing a premature stop codon at Env position 46. Three days later, cells were washed in blocking buffer (BB) composed of a tris-saline (TS) buffer (140 mM NaCl, 1.8 mM CaCl_2_, 1 mM MgCl_2_, and 25 mM Tris [pH 7.5]) supplemented with 3% bovine serum albumin and 1.1% skim milk. Cells were then incubated for 45 min at room temperature in BB that contains the antibodies at the following concentrations: 17 b and F105 at 5 μg/mL; 39 F and 10E8 at 2 μg/mL; CD4-Ig, N6, 447–52D, 35O22, PGT145 and 2G12 at 1 μg/mL. Cells were then washed 6 times with BB and incubated with a horseradish peroxidase-conjugated goat anti-human IgG for 1 h at room temperature. Cells were subsequently washed six times with BB and six times with TS buffer. Antibody binding was measured by chemiluminescence using 35 μL per well of a 1:1 mix of SuperSignal West Pico chemiluminescent peroxide and luminol enhancer solutions (Thermo Scientific) supplemented with 150 mM NaCl on a Synergy H1 microplate reader. To correct antibody binding values to the expression level of each Env, we also measured in each experiment their recognition by antibody 2G12 that targets an exposed epitope on the high-mannose patch of gp120.^95^ The antibody-to-2G12 binding ratio was calculated for each Env variant, and the value expressed as a fraction of this ratio measured for the wild-type AD8 Env. Binding assays were conducted at least three times in independent experiments, each with three replicate samples. Standard error values were used to describe the variability in the data.

#### Saturation mutagenesis to determine effects on Env fitness and resistance to TMR

To introduce a degenerate codon that encodes for all 20 amino acids (and one stop codon), we used primers that contain the trinucleotide sequence NNK at position 375 or 426 (see schematic of approach in Figure S12). N represents any nucleotide, and K represents G or T. The NNK-containing primers were used to amplify an Env segment using as template the proviral vector pNL4–3 that encodes for the Env of strain AD8 with PrimeSTAR Max DNA Polymerase (Takara). This fragment was then ligated to a second fragment by overlapping PCR to generate a combined fragment that spans the entire *env* gene. This product was then cloned into the env-deleted pNLAD8 vector (GenBank ID PV345784) using In-Fusion assembly (Takara), and the product transformed into Zymo Mix&Go DH5α chemically competent cells. At least 350 colonies for each library were collected and pooled, and plasmids were purified using ZR Plasmid Miniprep-Classic. Two micrograms of the provirus library were then used to transfect 1 × 10^6^ HEK 293 T cells cultured in 6-well-plate-wells using JetPrime reagent. The medium was changed after 8 h, and 48-h after transfection the virus was harvested, passed through 0.45 μm filters and treated with 100 U/mL DNase-1 (Roche) for 30 min at 37°C. Samples were then divided into aliquots, snap-frozen using dry ice immersed in ethanol, and stored at −80°C until use. Virus titers were determined by plaque assay after a 96-h infection of TZM-GFP cells (BEI Resources, HRP-20041, contributed by David G. Russell and David W. Gludish) in DMEM/FCS supplemented with 20 μg/mL DEAE dextran.

To eliminate mixed-allele virions from the samples, the above virus libraries were used to infect 2 × 10^6^ A3R5.7 acute lymphoblastic leukemia T cells in 2 mL at an MOI of 0.003 in RPMI/FCS supplemented with 20 μg/mL DEAE dextran. Cells were resuspended in 4 mL fresh culture medium 24 h after infection, and 2 days later the virus was harvested and filtered as above. Titers were determined using TZM-bl cells and the samples used to infect a culture of 1 × 10^6^ A3R5.7 cells in RPMI/FCS supplemented with 40 μg/mL DEAE dextran at an MOI of 0.05. These infections were performed in the absence or presence of 250 nM TMR. At 16 h post-infection, cells were pelleted and non-integrated viral DNA was purified using QIAprep Spin Miniprep kit (Qiagen). A 900-bp region encompassing position 375 and 426 was then amplified using PrimeSTAR Max DNA Polymerase. For each biological replicate, an initial 30-cycle amplification was performed in triplicate, the samples pooled, and gel-purified using Zymoclean Gel DNA Recovery Kit (Zymo). If necessary, an additional 8–12 rounds of PCR were performed to obtain sufficient sample for sequencing, which was purified using the QIAquick PCR Purification Kit (Qiagen). To sequence the input virus used for the second round of A3R5.7 cell infection, viral RNA was purified using Quick RNA Viral Kit (Zymo), reverse transcribed using SuperScript IV (Invitrogen), and PCR amplified using the same primers used with the purified product from the infected cells. Finally, all samples were sequenced by Oxford Nanopore Technology (PlasmidSaurus), which provided an average count of 5,000 reads per sample.

### QUANTIFICATION AND STATISTICAL ANALYSIS

#### Processing and analysis of sequence data

Plasma samples from BRIGHTE trial participants were collected before and during treatment and tested for viral loads and CD4 counts. In addition, some samples were analyzed using the Monogram Biosciences PhenoSense Entry and HIV Envelope NGS Sequencing Assays (Figure S13). Nucleotide and amino acid sequences for 580 samples from 371 subjects were available for analysis. TMR resistance values were available for 570 of these samples. Multiple Env positions contained ambiguous nucleotide or amino acid designations, which reflect the presence of more than one sequenced variant in the donor. To align such sequences, we initially demultiplexed the variants by retaining for each position the amino acid or nucleotide found in the clade B ancestral sequence, or, if not present, the first variant listed at that position. We then used a custom Python code to run MAFFT 7.520 for alignment.^88^ Sequences were then used to determine their clade associations using the Recombinant Identification Program (RIP) tool V3.0.^89^ Sequence variability information was subsequently reintegrated into the samples to allow more than one form at each position, and sequences trimmed to the subset of 856 positions according to HXBc2 numbering.^43^ For all unpaired T-tests and mixed effects models, statistical significance was denoted as: *, *p* < 0.05; **, *p* < 0.005; ***, *p* < 0.0005.

#### The Single-Env dataset and transformation of TMR resistance values

We also used a previously published dataset composed of 208 Envs from diverse clades, each associated with an amino acid sequence and TMR resistance value measured *in vitro*.^38^ Accession numbers (listed in Data S3) were used to download the sequences from the Los Alamos National Lab (LANL) database,^96^ which were then aligned and processed as above. TMR IC_50_ values for the Single-Env set were measured using an in-house assay,^38^ whereas the BRIGHTE trial samples were analyzed using the Monogram Biosciences Entry assay. To allow us to combine the datasets, we converted the Single-Env set values to a distribution similar to that of the pre-treatment samples from the BRIGHTE trial (Figure S3A). For this purpose, we first examined the distribution type of the BRIGHTE and Single-Env datasets, including Beta, Lognormal, Exponential, Gamma, and Pareto. Based on the sum of squared errors (SSE) metric, both datasets conformed best to a Beta Distribution. We then converted the Single-Env set IC_50_ values to the distribution of the pre-treatment BRIGHTE samples using a probability integral transform approach. In brief, we first mapped the Single-Env set values to their cumulative probabilities, then applied the inverse cumulative distribution function of the BRIGHTE Beta distribution, and finally rescaled to the target support, ensuring the values follow the BRIGHTE distribution while preserving their rank ordering.

#### Classification algorithms to estimate env resistance to TMR by sequence

We evaluated different classification algorithms for their performance to estimate TMR resistance by amino acid sequence. For these evaluations, as well as for hyperparameter tuning, we defined an IC_50_ of 50 nM TMR as the threshold to distinguish between sensitive and resistant samples. As input for the model, we first used the amino acids at the 856 positions of Env according to the HxBC2 numbering system. To account for sequence ambiguity in the BRIGHTE data, we used one-hot encoding to convert each position into binary features representing the absence (0) or presence (1) of each amino acid in the sample. In addition, given that some subjects had multiple samples analyzed (before and after treatment), we used a group-stratified 5-fold cross validation approach, which ensured that all sequences of each subject are assigned to either the training or test folds.

Following preliminary testing of different classification algorithms, we pursued Extreme Gradient Boosting (XGBoost) from the *xgboost* package in Python,^44^ for its high performance across several key metrics (Figure S2A). Parameter fine-tuning was performed through grid search in Python to enhance the model’s predictive capacity. The following hyperparameters were tuned: The number of estimators was set to 100, 150, 200, 250 and 300 trees; learning rate (η) was set to 0.1, 0.2 and 0.3; maximum depth of trees was set to 3, 5 and 8; Lambda Regularization was 1, 2, and 3; and the fraction of features and samples used by the algorithm was 1. The AUC was used as the objective function for optimization. Classification metrics were calculated using the *metrics* module from the *sklearn* library.^97^

To avoid overfitting the data, we used a 5-fold cross validation approach and reported variability among the folds. For further validation, we trained a model on the BRIGHTE dataset (570 samples) and tested it on the Single-Env dataset (208 samples). This dataset was composed of Envs from a broader range of HIV-1 clades (compare clade distribution in Figures 1C and S1B). The high performance of the model with the Single-Env dataset suggested limited overfitting of the data (Figure S3).

#### Gradient Boosting Regressor to identify env features that impact resistance to TMR

To identify the sequence features that contribute to TMR resistance, we used the Gradient Boosting Regressor (GBR) algorithm. The algorithm was trained on the combined dataset of 570 BRIGHTE and 208 Single-Env samples. Amino acid sequences were used as the input feature set, and the log_10_-transformed TMR IC_50_ values as the response variable. To prepare the sequences, we first excluded Env positions with a minimal distance greater than 7.5 Å from any TMR atom on the TMR-liganded structure of Env (PDB ID 5U7O), resulting in 56 remaining positions (Table S1). Next, we applied one-hot encoding to convert the amino acid occupancy at each position into binary features. Of the 243 remaining features, nine exhibited no variation and were excluded. To further reduce dimensionality and mitigate multicollinearity, we dropped the least frequent feature at each position that meets two criteria: (i) The position contains at least two unique AA variants in the dataset, and (ii) The mutation is included in the feature set tested by the probabilistic approach (see below). This reduced the feature set to 129.

The GBR model was trained using mean squared error (MSE) as the objective function for optimization with the Friedman improvement score as the criterion for measuring split quality, and a learning rate (*η*) value of 0.1. A k-fold nested grouped cross-validation strategy was used with *k* = 10 for the outer folds and *k* = 5 for the inner folds. This nested structure separates the parameter optimization process from the model assessment with the inner cross-validation loop for hyperparameter tuning, and the outer loop for model evaluation. The grouping strategy ensured that sequences from the same subject appeared in either the training or test fold, but not both. Hyperparameters of the GBR model were optimized via grid search, including the number of estimators (20, 50, or 100) and the maximum depth of individual regression estimators (3, 5, 10, or full tree). Predicted IC_50_ values that exceeded 5 μM were capped at the maximal allowable value of 5 μM TMR. To estimate the contribution of features to the model’s predictions, we used the Shapley Additive Explanations (SHAP) value.^45^ SHAP values quantitatively describe the impact of each feature on the model’s performance to predict the outcome, including their magnitude, direction and distribution.

#### Probabilistic approach to identify low-prevalence mutations that impact TMR resistance

The occurrence of mutations at a low frequency in the dataset limits the ability of algorithms to model their effect. To estimate their impact, we implemented a separate procedure. Mutations included in this set were defined by three criteria: (i) They must appear in at least two unique sequences, (ii) They must not appear in more than 5% of all sequences, and (iii) The side chain of the position must be located within 7.5 Å of the TMR molecule. This filtering process resulted in 107 mutations. If a mutation appeared in more than one sample of a BRIGHTE participant, the average value of the log_10_(IC_50_) was used.

A prioritization algorithm was used to rank these mutations by their potential impact. For each of the 107 mutations, described as amino acid *a* and position *p*, we modeled the log10-converted IC50 values of samples containing that mutation using a normal distribution *X*^*a*,*p*^, with average *μ* and variance *σ*^2^. We assume that *X*^*a*,*p*^ corresponds with the distribution of their effects on the greater population of HIV-1 strains (*Y*^*a*,*p*^). To compare between the 107 mutations, we randomly sampled with replacement the distribution *X*^*a*,*p*^ of each mutation. This value was compared with the randomly sampled value from all other 106 mutations and ranked (highest value received the lowest rank). This process was repeated 10,000 times, and the average ranks for all 107 mutations across all iterations were calculated. Figure S4D summarizes the top features identified by this approach.

#### Statistical analyses

Statistical analyses were performed in Python. Associations between REM emergence rates and Env fitness features (functional fitness, stability at 37°C, and immune fitness proxies) were assessed using Spearman rank correlation tests, and corresponding P-values were reported. Quantification of mutation enrichment in the 65 participants who developed resistance on therapy is described in the following section. For sequence-based inference, model discrimination was evaluated using group-stratified cross-validation (to avoid patient-level leakage) and nested grouped cross-validation for hyperparameter tuning. Classification performance was summarized by the area under the ROC curve (AUC) using an IC_50_ threshold of 50 nM for defining resistance.

For statistical analyses of variant sensitivities to the plasma, given that two samples from different donors were used, we applied a mixed effects model. The two plasma samples were tested in two independent experiments, each performed with three technical replicates. To assess the effect of Env type, we used the log_10_-transformed plasma dilution required for 50% inhibition. A linear mixed-effects model was applied, with virus genotype (wild-type versus mutant) treated as the fixed effect, and plasma donor and experiment included as random effects.

#### Analysis of mutation enrichment in the escape group after treatment

We compared the frequency of all 59 suspected mutations in the samples collected before and during treatment in the escape group. The escape group was defined as participants for whom all pre-treatment samples had IC_50_ values lower than 50 nM TMR, and at least one on-treatment sample with an IC_50_ greater than 50 nM. To determine enrichment of mutations after treatment in these 65 participants, we tested the null hypothesis that the frequency of the mutations in the pre-treatment samples was equal to or greater than their frequency in the post-treatment samples. Briefly, for each subject we merged all amino acids at each position into a single pre-treatment sample or post-treatment sample. We then calculated for each of the 59 suspected mutations the ratio between the mutation frequency in the post-treatment samples relative to the pre-treatment samples. If a participant contained a mutation in both pre- and post-treatment samples, the individual was excluded from the analysis of that mutation. Time point identifiers were then permuted 10,000 times and for each iteration the ratio was calculated. To avoid dividing by zero, a small constant value was added to the denominator. The fraction of iterations in which the ratio for the permuted data was the same or larger than the non-permuted data was applied as the P-value for this one-sided test.

#### Rate of independent substitution events in HIV-1 clade B

We calculated the emergence frequency of the 18 REMs in HIV-1 clade B. To this end, we downloaded Env amino acid sequences from the LANL database^96^ and aligned them to the HXBc2 strain. All sequences that contain any ambiguity or stop codons were removed, as well as sequences within 0.05 amino acid substitutions per site from any other sequence. The resulting dataset consisted of 2,535 Env sequences. We note that while no information was associated with these sequences to indicate lack of fostemsavir treatment of the hosts, the sample collection dates (99.2% collected before FDA approval of fostemsavir) and the sources of the remaining samples render the likelihood of such a coincidence negligible. Phylogenetic relationships between sequences were calculated using FastTree^90^ on the Galaxy platform,^91^ and the tree was divided into sublineages comprising 80 to 150 sequences each using the Depth-First Search algorithm.^98^ All sublineages were processed using HyPhy SLAC^92^ on Galaxy to infer the most likely ancestral sequence. A custom python code was then used to calculate the rate of independent mutation events at each Env position using the results of SLAC as an input. In brief, each subgroup tree was recursively searched to determine all substitution events relative to the prior inferred node. A subgroup was ignored if the majority variant differed from the clade consensus sequence. The rate of independent emergence events of for each amino acid was calculated as the ratio between the number of emergence events and the total number of nodes. Daughter nodes of inferred substitution events were excluded from this count unless a new substitution event occurred. The code is available through our GitHub repository at https://github.com/haimlab/Independent_mutation_Tree.

#### Calculation of amino acid preferences in deep mutational scanning experiments

Sequence data (in fastq format) were used to calculate amino acid preferences at positions 375 and 426 in the absence and presence of Temsavir based on the method described by Haddox and colleagues.^52^ This approach calculates the relative frequency of each amino acid in viral DNA isolated from cells infected by the virus library relative to their frequencies in the virus library used for infection. To determine if calculations should be corrected for the error rate in the sequencing reactions, we first examined the incorrect variant calls at the two positions by sequencing three samples infected by the wild-type virus. The average frequency of minority variants across the six samples was 0.13% (standard deviation 0.12%). As such, we performed subsequent calculations in an error-agnostic manner.

To calculate the enrichment ratio (ϕ) for each amino acid a at position p, we used:

$$\phi_{p,a}=\frac{f_{p,a}^{\mathrm{cell}}/f_{p,wt(p)}^{\mathrm{cell}}}{f_{p,a}^{\mathrm{virus}}/f_{p,wt(p)}^{\mathrm{virus}}}$$

where $f_{p,a}^{\mathrm{cell}}$ and $f_{p,wt(p)}^{\mathrm{cell}}$ are the frequencies in the cell lysate of amino acid a or the wild-type (wt) amino acid for position p, and $f_{p,a}^{\mathrm{virus}}$ and $f_{p,wt(p)}^{\mathrm{virus}}$ are the frequencies of these forms in the input virus sample used for infection. Finally, we calculated the amino acid preference (π) for each amino acid as:

$$\pi_{p,a}=\frac{\phi_{p,a}}{\sum_{a^{\prime }}\phi_{p,a^{\prime }}}$$

where the $\sum_{a^{\prime }}\phi_{p,a^{\prime }}$ is the sum of enrichments for all amino acids $a^{\prime }$ at position p.

A complete software package to calculate amino acid preferences is available through our Github repository at https://github.com/haimlab/EZDMS.

#### Algorithms to estimate appearance and persistence of REMs

To evaluate the contribution of the different variables to the observed mutational profiles in treated individuals, we designed two algorithms (see flowcharts in Figures S10B and S10C). In both, mutation appearance and persistence were modeled as stochastic events with mutation-specific probabilities. The first algorithm calculated for each of the 18 REMs individually the probability of appearance based on the number and type of nucleotide changes required from the initial trinucleotide sequence. To test the probability for REM appearance in clade B, the algorithm was initiated with the trinucleotide sequence at the Env position in the clade B consensus sequence. To test the probability for REM appearance in the 65 participants of the escape group, the algorithm was initiated with the trinucleotide sequence in the pre-treatment samples of the 65 escape group participants. One of the three nucleotide sites was selected randomly, and a nucleotide substitution was randomly introduced at that site based on the transition and transversion likelihoods determined by Martinez Del Rio et al.,^15^ (see values in Figure S10A). Up to two consecutive mutations were allowed. If the desired REM was acquired within the two attempts, a success event was recorded. For each REM, the algorithm was repeated 1,000 times for the clade B consensus sequence, and 1,000 times for each escape group participant. The fraction of success events of the 1,000 iterations in each participant was defined as their probability for mutation appearance.

The second algorithm (Figure S10C) introduced an additional module to the mutation appearance step, which aimed to capture the ability of each REM to persist in the host. It was based on the *in vitro* measured effects of the REMs on: (i) Infectivity, (ii) Functional stability at 37°C, and (iii) Resistance to non-neutralizing plasma. The three values measured for each REM were expressed as a fraction of the wild-type AD8 Env values, and their product was defined as the Combined Fitness value of each REM. For each success event from the appearance module, persistence was determined by a random process, in which the Combined Fitness value was used as the probability of the REM to persist. If the mutation persisted, a success event was recorded. The fraction of success events of the 1,000 iterations was defined as the probability for REM emergence. Performance was evaluated by compiling the probability values for the 18 REMs in the 65 participants of the escape group, which were compared with their outcomes: the absence (0) or presence (1) of REM emergence in the participant after treatment. Performance was measured using the AUC metric. The code for the above algorithm can be found on our Github repository at https://github.com/haimlab/REM-Emergence-Modeling.

## Supplementary Material

1

2

4

5

6

7

8

9

10

11

SUPPLEMENTAL INFORMATION

Supplemental information can be found online at https://doi.org/10.1016/j.celrep.2026.117558.

## Figures and Tables

**KEY RESOURCES TABLE:** 

REAGENT or RESOURCE	SOURCE	IDENTIFIER

Antibodies		

Monoclonal Anti-Human Immunodeficiency Virus Type 1 (HIV-1) gp120 Protein, Clone 17 b	NIH-ARP/BEI Resources	Cat#ARP-4091; Lot #s 170396 & 150329
Anti-Human Immunodeficiency Virus (HIV)-1 gp120 Monoclonal Antibody (F105)	NIH-ARP/BEI Resources	Cat#ARP-857; Lot#180246
Monoclonal Anti-Human Immunodeficiency Virus Type 1 (HIV-1) gp120 (39 F)	NIH-ARP/BEI Resources	Cat#ARP-11437; Lot#130014
Anti-Human Immunodeficiency Virus (HIV)-1 gp41 Monoclonal Antibody (10E8)	NIH-ARP/BEI Resources	Cat#ARP-12294; Lot#170395
Human CD4-Ig Recombinant Protein	NIH-ARP/BEI Resources	Cat#ARP-13058; Lot#170155
Recombinant Anti-CD4-Binding Site Human IgG1 Kappa Monoclonal Antibody (Clone N6)	Syd Labs	Cat#PA007171; Lot#230825-1
Anti-Human Immunodeficiency Virus (HIV)-1 gp120 Monoclonal Antibody (447-52D)	NIH-ARP/BEI Resources	Cat#ARP-4030; Lot#160024
Monoclonal Anti-Human Immunodeficiency Virus Type 1 (HIV-1) gp41/gp120 Glycoprotein (35022)	NIH-ARP/BEI Resources	Cat#ARP-12586; Lot#150359
Monoclonal Anti-Human Immunodeficiency Virus Type 1 (HIV-1) gp120 Protein (PGT145)	NIH-ARP/BEI Resources	Cat#ARP-12703; Lot#170023
Monoclonal Anti-Human Immunodeficiency Virus Type 1 (HIV-1) gp120 (2G12)	NIH-ARP/BEI Resources	Cat#ARP-1476
Horseradish peroxidase-conjugated goat anti-human IgG	Invitrogen	Cat#PI31412

Bacterial and virus strains		

*Mix & Go!* Competent Cells - DH5 Alpha	Zymo Research	Cat#T3007

Biological samples		

Plasma from HIV-1 infected individuals	The University of Iowa	N/A

Chemicals, peptides, and recombinant proteins		

Temsavir	MedChemExpress	Cat#HY-15440; BMS-626529
PrimeSTAR^®^ Max DNA Polymerase 2x Premix	Takara Bio	Cat#R045A
OneTaq^®^ Quick-Load^®^ 2X Master Mix with Standard Buffer	New England Biolabs	Cat#M0486S
DpnI	New England Biolabs	Cat#R0176S
rCutSmart^™^ Buffer	New England Biolabs	Cat#B6004S
In-Fusion^®^ Snap Assembly Master Mix	Takara Bio	Cat# 638948
jetPRIME^®^ Transfection Reagent & Buffer	Polyplus/Sartorius	Cat#101000015
Passive Lysis 5x Buffer	Promega	Cat#E1941
Fetal Bovine Serum	Avantor	Cat#89510-186
Penicillin-Streptomycin	Gibco	Cat#15140
G418 Disulfate Solution	IBI Scientific	Cat#IB02060; CAS: 108321-42-2
Hygromycin B	Invitrogen	Cat#10687010
D-Luciferin	Syd Labs	Cat#MB000102-R70170
TaqMan^™^ Gene Expression Master Mix	Applied Biosystems (Thermo Fisher)	Cat#4369016
Ribolock RNase Inhibitor	Thermo Scientific	Cat#EO0381
HIV-1 BH10 Reverse Transcriptase Recombinant Protein	NIH-ARP/BEI Resources	Cat#ARP-12583
Triton^®^ X-100	Fisher Scientific	Cat#BP151
UltraPure^™^ 1 M Tris-HCl pH 7.5	Invitrogen	Cat#15567-027
Bovine serum albumin	Research Products International	Cat#A30075; CAS: 9048-46-8
SuperSignal^™^ West Pico PLUS Luminol/Enhancer, SuperSignal^™^ West Pico PLUS Stable Peroxide	Thermo Scientific	Cat#34577
DNase-I	Roche	Cat#10104159001
DEAE Dextran	Sigma-Aldrich	Cat#D9885
RiboLock RNase Inhibitor	Thermo Scientific	Cat#EO0381
dNTP mix	Invitrogen	Part#100011241
Random Primers	Invitrogen	Cat#48190011
RNaseOUT^™^ Recombinant Ribonuclease Inhibitor	Invitrogen	Cat#10777019
SuperScript^™^ IV Reverse Transcriptase, 5x SSIV Buffer, 0.1 M DTT	Invitrogen	Cat#18090010

Critical commercial assays		

PhenoSense^®^ Entry and HIV Envelope NGS Sequencing Assay	Monogram Biosciences	N/A
QIAquick^®^ PCR Purification Kit	Qiagen	Cat#28106
Zymoclean^™^ Gel DNA Recovery Kit	Zymo Research	Cat#D4007
ZR Plasmid Miniprep - Classic	Zymo Research	Cat#D4015
Qiaprep Spin Miniprep Kit	Qiagen	Cat#27104
NucleoBond^®^ Xtra Midi	Macherey-Nagel	Cat#740410
Quick-RNA^™^ Viral Kit	Zymo Research	Cat#R1034

Deposited data		

BRIGHTE amino acid sequences and TMR resistance values	This paper	Data File S1
Supplemental dataset amino acid sequences and TMR resistance values	This paper; Pancera et al.^30^	Data File S3

Experimental models: Cell lines		

Human: HEK293T cells	ATCC	RRID: CVCL_0063
Dog: Cf2Th CD4+ CCR5+ cells	Joseph Sodroski	N/A
Dog: Cf2Th CCR5+ cells	Joseph Sodroski	RRID: CVCL_1G50
Human: HOS cells	ATCC	RRID: CVCL_0312
Human: TZM-GFP	BEI Resources	Cat#HRP-20041
Human: A3R5.7	BEI Resources	Cat#ARP-12386; RRID: CVCL_S540

Oligonucleotides		

See all primers used in this study in Data File S9	This paper	N/A
MS2 Bacteriophage RNA	Roche	Cat#10165948001

Recombinant DNA		

Plasmid: psvIII AD8	Joseph Sodroski	N/A
Plasmid: JRFL Envelope, cloned into psVIII	Joseph Sodroski	N/A
Plasmid: QH0692 Envelope, cloned into pCDNA3.1 (GenBank accession code AY835439)	BEI Resources	ARP-11018
Plasmid: WEAUd15 Envelope, cloned into pCDNA3.1 (GenBank accession code EU289202)	BEI Resources	ARP-11578
Plasmid: 700010040 Envelope, cloned into pCDNA3.1 (GenBank accession code EU289193)	BEI Resources	ARP-11569
Plasmid: pCMVΔP1 ΔenvpA	Joseph Sodroski	N/A
Plasmid: pHIvec2.luc	Joseph Sodroski	N/A
Plasmid: pRev	Joseph Sodroski	N/A
Plasmid: Tat	Joseph Sodroski	N/A
Plasmid: HIV-1 vector pNL(AD8); GenBank accession code GenBank ID: PV345784.1	BEI Resources	HRP-11346

Software and algorithms		

MAFFT 7.520	Katoh and Standley^88^	https://mafft.cbrc.jp/alignment/server/index.html
Recombinant Identification Program (RIP) 3.0	Siepel et al.^89^	https://www.hiv.lanl.gov/content/sequence/RIP/RIP.html
FastTree 2.2	Price et al.^90^	https://github.com/morgannprice/fasttree
Galaxy platform on the European server	Galaxy^91^	https://usegalaxy.eu/
HyPhy SLAC v2.5.97	Kosakovsky et al.^92^	https://github.com/veg/hyphy
Machine learning algorithms: scikit-learn library (v1.7); XGBoost: xgboost library	Chen and Guestrin^44^	https://github.com/dmlc/xgboost
Python code for calculating independent mutation rate	This paper	https://github.com/haimlab/Independent_mutation_Tree
Simulation of mutation emergence	This paper	https://github.com/haimlab/REM-Emergence-Modeling
EZ-DMS	This paper	https://github.com/haimlab/EZDMS

Other		

Custom probe for qPCR: 6[FAM] CGAGACGCTACCATGGCTATCGCTGTAG [TAMsp]	Integrated DNA Technologies (IDT)	N/A

## Highlights

- Systematic screen maps multiple HIV-1 Env mutations conferring resistance to temsavir
- *In vivo*, resistance converges on a limited set of pathways
- On-treatment frequencies parallel mutation rates in untreated individuals
- Emergence is governed by mutation probability, virus fitness, and immune constraints
