# Supplementary material for: Comprehensive mapping of the virus and host factors that guide the paths of HIV-1 escape from a therapeutic
Source: Cell Rep. Author manuscript; Available in PMC 2026 Sep 11. (PMC13564207; doi:10.1016/j.celrep.2026.117558)
Supplement: Supplementary file 1 [file NIHMS2199924-supplement-1.pdf]

Cell Reports, Volume *45*

Supplemental information

Comprehensive mapping of the virus and host factors that guide the paths of HIV-1 escape from a therapeutic

Aaron N. Gillman, Cassian M. Birler, Rohith Rao Vujjini, Mohammad Fili, Samuel A. McCarthy-Potter, Wilson Chen, Madeline M. Broghammer, Guiping Hu, Margaret J. Gartland, Manyu Prakash, Annika Helverson, Grant G. Brown, and Hillel Haim

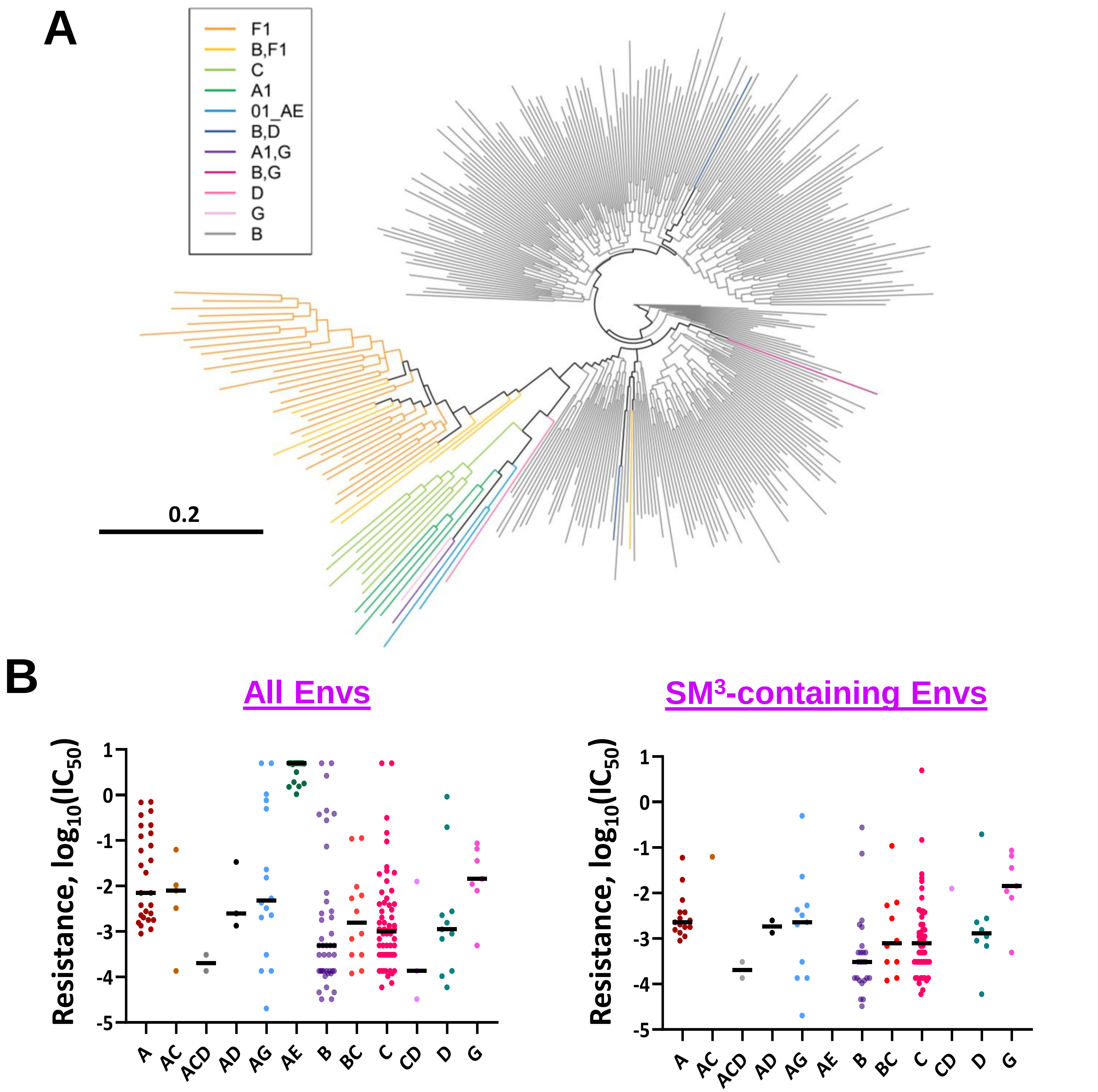

Supplemental Figure S1. Clade distribution of the BRIGHTE trial (A) and Single-Env datasets. Related to Fig 1.

(A) Phylogenetic tree based on nucleotide sequences of Env from pre-treatment samples of 358 BRIGHTE trial participants. A single sequence was used for each participant. Clade associations were inferred using the RIP tool.

(B) Clade distribution of the Single-Env dataset. Resistance values for the 208 Envs of the Single-Env dataset (as reported in Pancera et al., 2017) and for the subset of samples that contains the TMR-sensitive SM^3 motif. Samples are grouped by their inferred clade associations as reported by the authors. Log_{10}-converted IC_{50} values in the two groups were compared using an F-test for the equality of variances (P-value < 10^{-6}). A similar analysis compared samples that contain the SM^3 motif with those lacking it (P-value < 10^{6}).

A

Model	AUC	Accuracy	F1 Score	Precision	Sensitivity	Specificity
**XGBoost**	0.95 (0.02)	0.90 (0.03)	0.82 (0.03)	0.85 (0.06)	0.79 (0.05)	0.94 (0.03)
**Gradient Boosting**	0.94 (0.02)	0.89 (0.03)	0.81 (0.04)	0.86 (0.05)	0.77 (0.06)	0.95 (0.02)
**Logistic Regression**	0.92 (0.01)	0.88 (0.02)	0.80 (0.02)	0.83 (0.06)	0.76 (0.04)	0.92 (0.04)
**AdaBoost**	0.85 (0.04)	0.85 (0.03)	0.79 (0.05)	0.82 (0.05)	0.78 (0.09)	0.88 (0.03)
**Random Forest**	0.79 (0.02)	0.82 (0.02)	0.72 (0.03)	0.75 (0.04)	0.69 (0.04)	0.81 (0.01)
**SVM**	0.70 (0.02)	0.72 (0.03)	0.70 (0.03)	0.71 (0.04)	0.64 (0.02)	0.73 (0.03)

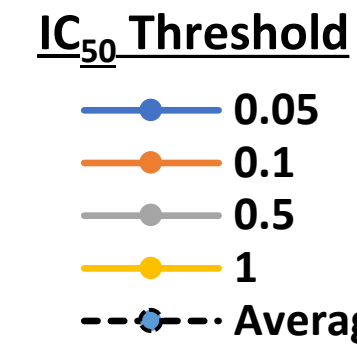

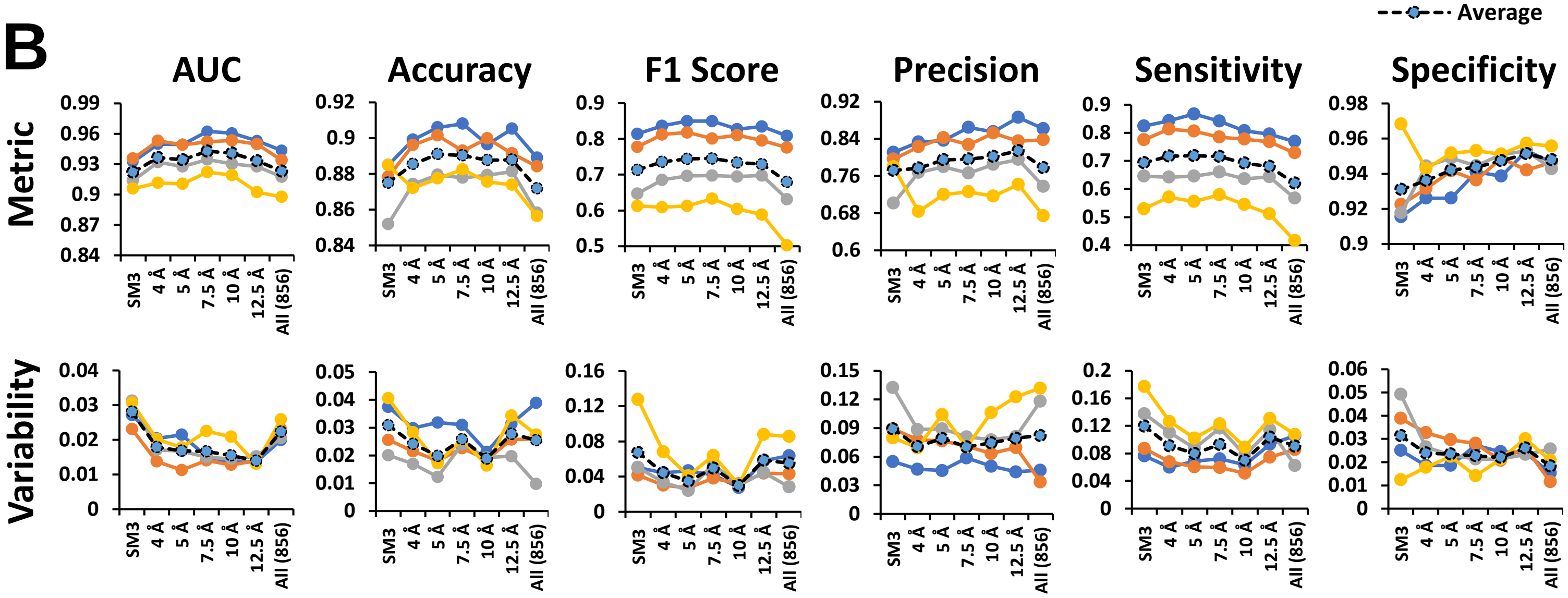

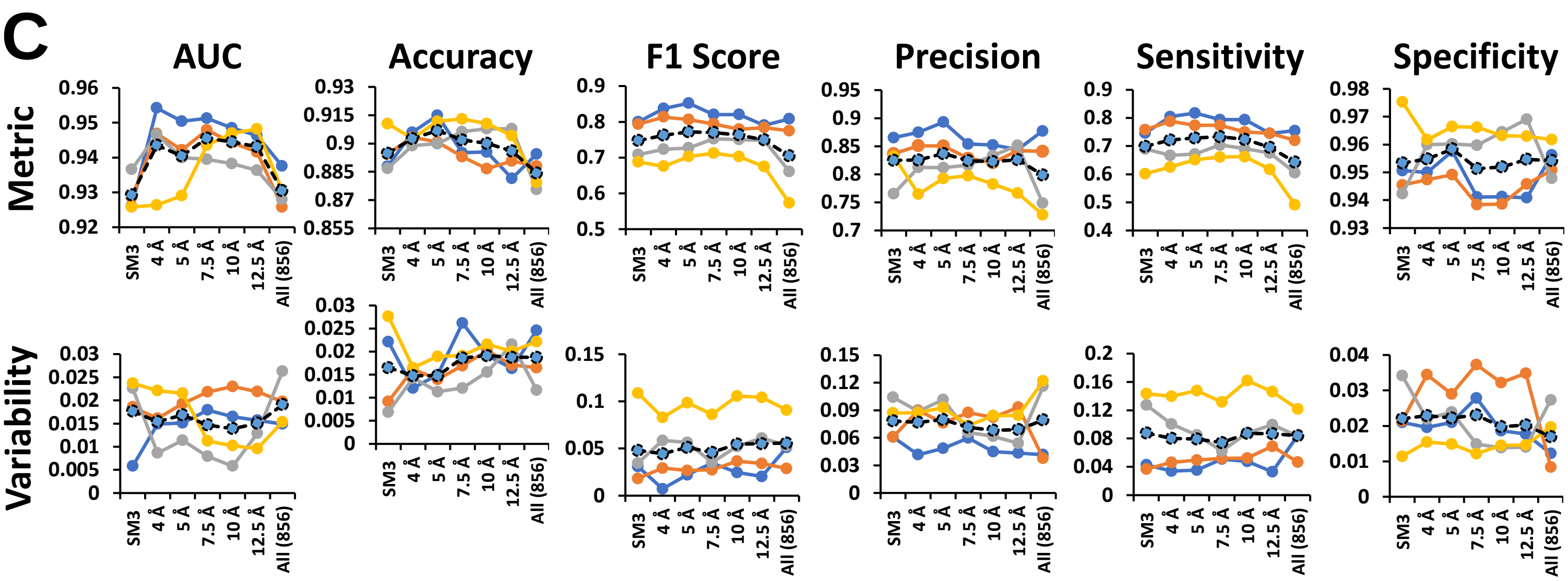

Supplemental Figure S2. Prediction of HIV-1 resistance to TMR using classification algorithms. Related to Fig 2.

(A) Performance of different classification-based algorithms to predict TMR resistance by sequence. Amino acid sequence data for all 856 positions of Env in the 570 samples from the BRIGHTE trial were used as input to the indicated learners to predict resistance. Resistance was defined as an IC_{50} value greater than 50 nM. Classification metrics are shown. Values in parentheses describe standard deviations for five-fold cross validation. AUC, area under the curve; SVM, Support Vector Machines.

(B,C) Classification metrics for prediction of resistance for the 570 BRIGHTE samples **(B)** or the 778 samples of the combined BRIGHTE and Single-Env datasets **(C)** using the XGBoost algorithm. As input, we used the amino acid sequences at positions within the indicated distances from the TMR molecule, the four SM^3 positions, or all 856 positions of Env. Mean metric values for the five folds are shown in the top graphs and the variability (in standard deviation) in the bottom graphs. Averages values for the different IC_{50} thresholds are shown as black dashed lines.

A

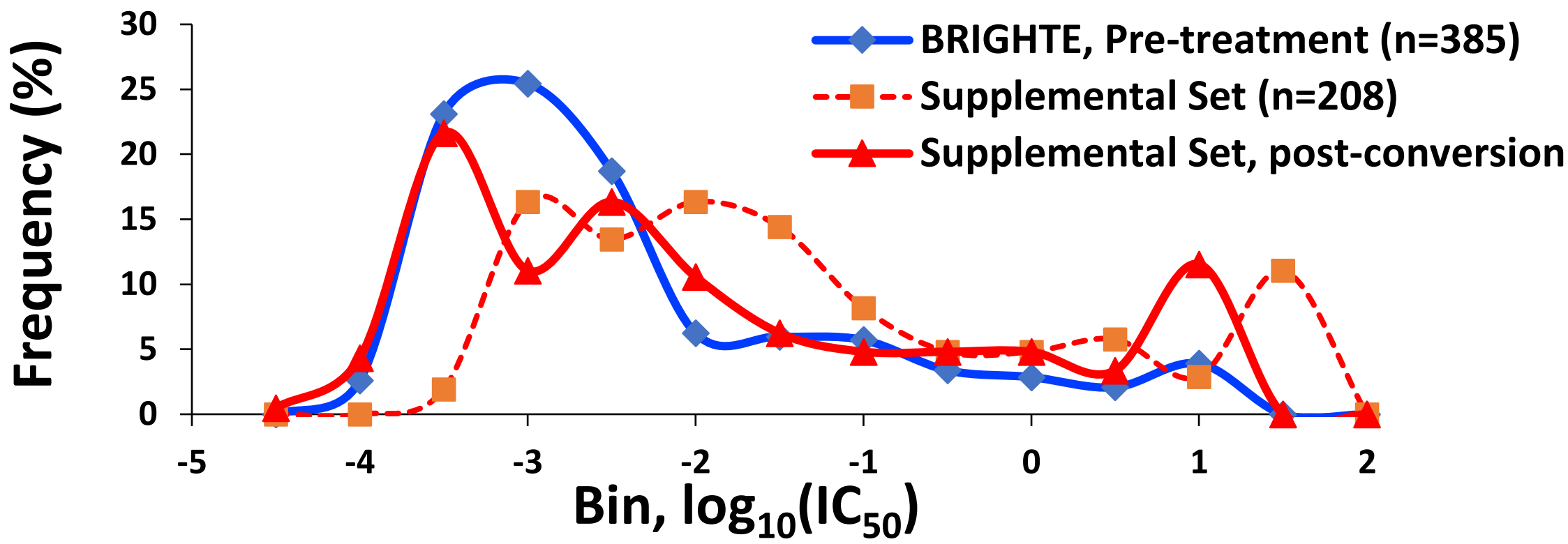

B

Model	AUC	Accuracy	F1 Score	Precision	Sensitivity	Specificity
XGBoost	0.94 (0.01)	0.89 (0.02)	0.81 (0.05)	0.88 (0.04)	0.75 (0.08)	0.96 (0.01)
Gradient Boosting	0.94 (0.01)	0.89 (0.31)	0.80 (0.04)	0.85 (0.42)	0.73 (0.07)	0.94 (0.02)
Logistic Regression	0.87 (0.03)	0.83 (0.01)	0.66 (0.05)	0.85 (0.04)	0.54 (0.06)	0.96 (0.01)
AdaBoost	0.89 (0.02)	0.86 (0.01)	0.75 (0.03)	0.81 (0.40)	0.71 (0.05)	0.93 (0.01)
Random Forest	0.77 (0.02)	0.77 (0.02)	0.54 (0.02)	0.78 (0.07)	0.42 (0.10)	0.80 (0.02)
SVM	0.81 (0.05)	0.76 (0.02)	0.68 (0.07)	0.58 (0.09)	0.68 (0.06)	0.85 (0.02)

C

Model	AUC	Accuracy	F1 Score	Precision	Sensitivity	Specificity
XGBoost	0.91	0.89	0.78	0.91	0.79	0.97
Gradient Boosting	0.88	0.88	0.78	0.84	0.72	0.95
Logistic Regression	0.88	0.84	0.74	0.69	0.79	0.86
AdaBoost	0.9	0.88	0.8	0.78	0.81	0.91
Random Forest	0.9	0.88	0.77	0.79	0.76	0.89
SVM	0.85	0.84	0.75	0.77	0.65	0.87

D

Model	AUC	Accuracy	F1 Score	Precision	Sensitivity	Specificity
XGBoost	0.90	0.86	0.84	0.86	0.86	0.89

E

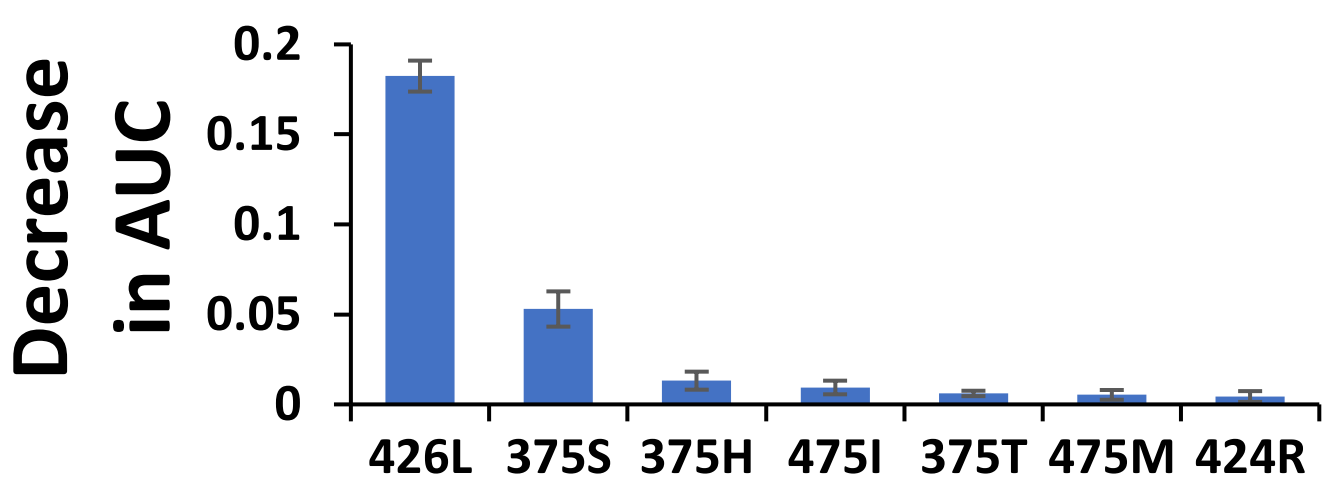

F

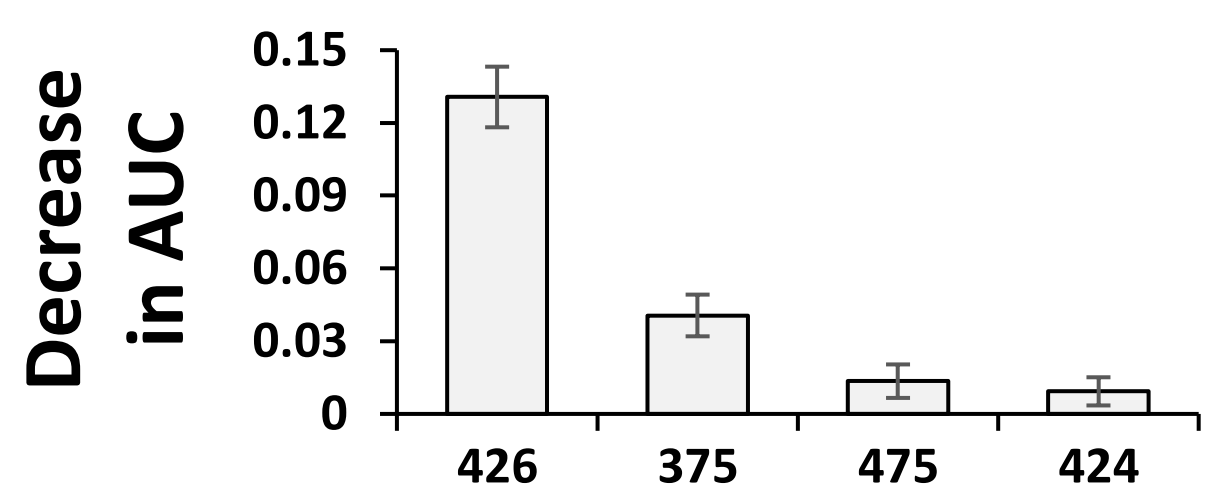

Supplemental Figure S3. **Prediction of TMR resistance using the combined BRIGHTE and Single-Env datasets.** Related to Fig 2.

(A) Normalization of IC_{50} values between datasets. TMR resistance values for the BRIGHTE trial samples and the Single-Env dataset were measured using different *in vitro* assays. As such, we normalized the IC_{50} values of the latter dataset to their distribution in the pre-treatment samples from the BRIGHTE trial (see Methods Section).

(B) Performance of different classification-based algorithms to predict TMR resistance by sequence, using as input the combined 778 samples from the BRIGHTE and Single-Env datasets. IC_{50} values for the latter group were normalized to the distribution of the former. Amino acids at the 856 positions of Env according to the HXBc2 numbering system were used as input for the model with a threshold of 50 nM to define resistance.

(C) Performance of the models to predict resistance of the 778 samples from the BRIGHTE and Single-Env datasets. The amino acid sequences at the 56 positions within 7.5 Å from the TMR molecule were used as input.

(D) Performance of an XGBoost model trained on 516 Envs from HIV-1 clade B (from the BRIGHTE and Single-Env datasets) and tested on 262 Envs from all other HIV-1 clades. All 856 positions of Env were used as input.

(E,F) Leave-one-out analysis to identify Env features associated with TMR resistance. The BRIGHTE and Single-Env datasets were combined and used to predict TMR resistance using XGBoost. A leave-one-out analysis was performed for **(E)** Each of the 5490 Env features (i.e., all residues represented in the dataset at all 856 Env positions), or **(F)** Each of the 856 positions of Env (i.e., all residues at that position). The test was performed 5 times and the average change in AUC upon feature or site exclusion was calculated. The top features are shown.

A

Metric	Value
MSE	0.63 ± 0.15[a]
MAE	0.57 ± 0.07
RMSE	0.26 ± 0.06
RMAE	0.42 ± 0.05

C

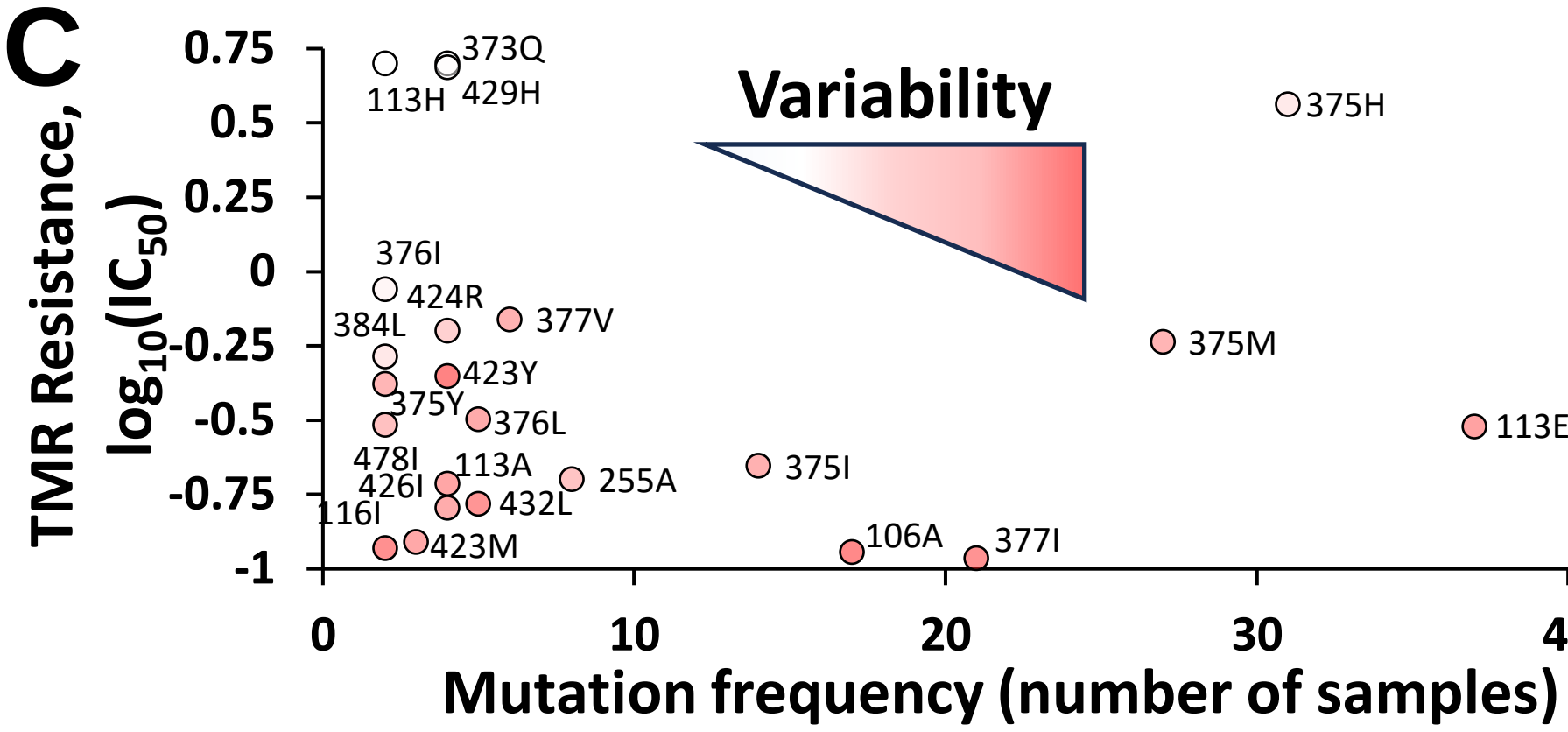

B

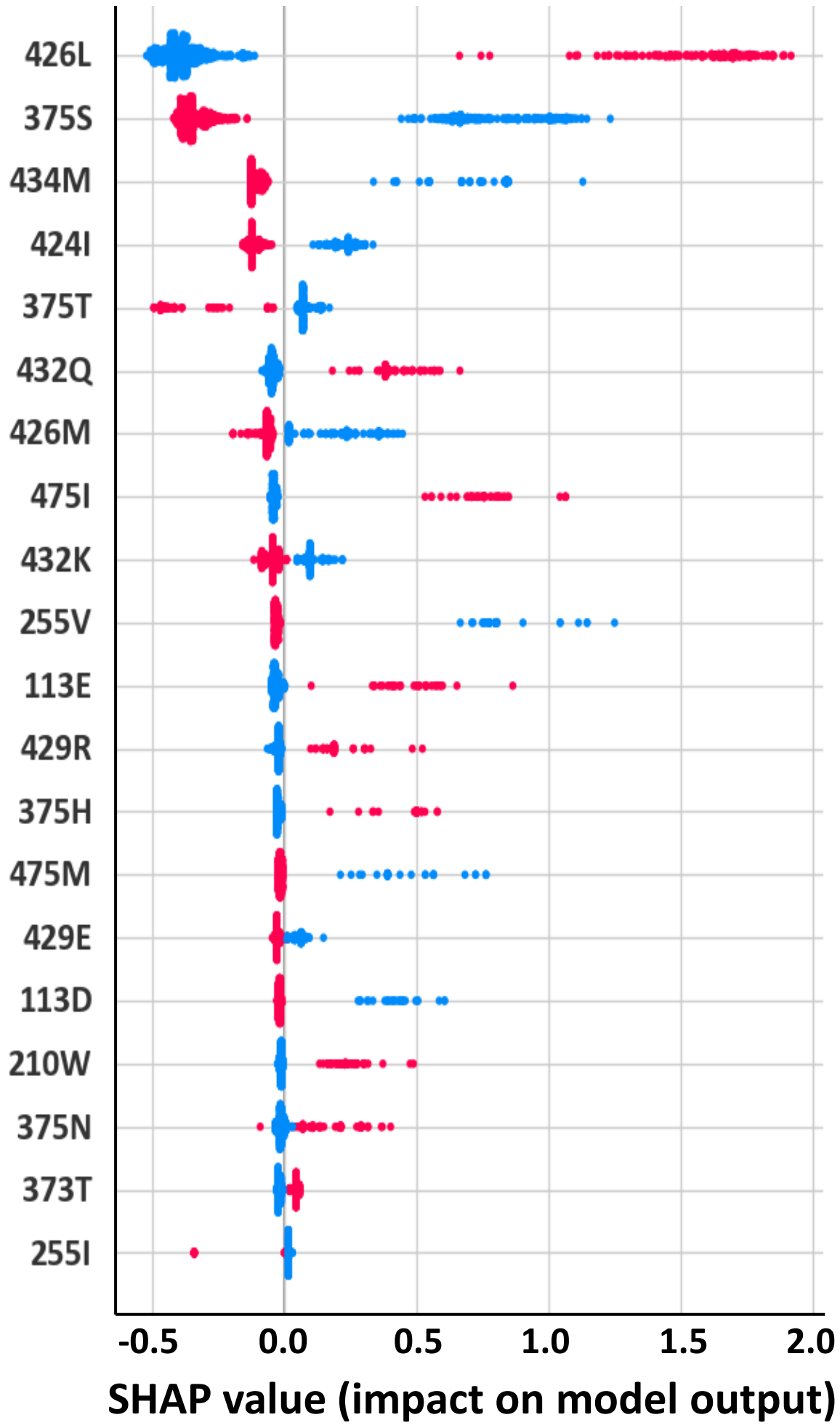

D

#	Feature	Avg. Rank	Distance (Å)	# of unique Seqs (n)	$\bar{x}$	s
1	113H	2.3	1.7	2	0.70	0.00
2	373Q	2.3	6.2	4	0.70	0.00
3	429H	3.5	7.0	4	0.69	0.02
4	375H	4.9	2.5	31	0.56	0.31
5	376I	9.4	3.2	2	-0.06	0.12
6	375M	12.2	2.5	27	-0.24	1.09
7	424R	12.2	2.9	4	-0.20	0.67
8	377V	12.3	5.0	6	-0.16	1.14
9	384L	13.1	3.5	2	-0.29	0.34
10	113E	17.1	1.7	37	-0.52	1.37
11	375Y	18.0	2.5	2	-0.38	1.08
12	376L	18.2	3.2	5	-0.50	1.23
13	423Y	18.9	6.3	4	-0.35	1.82
14	478I	19.5	6.9	2	-0.52	0.91
15	375I	20.0	2.5	14	-0.65	1.15
16	255A	20.8	2.3	8	-0.70	0.87
17	113A	23.5	1.7	4	-0.71	1.31
18	426I	24.6	3.6	4	-0.80	1.22
19	432L	25.6	2.5	5	-0.78	1.56

Supplemental Figure S4. Identification of Env features associated with TMR resistance. Related to Fig 2.

(A,B) Gradient boosting regressor to identify mutations associated with TMR resistance. The 778 samples of the BRIGHTE and Single-Env datasets were used to predict Env resistance by sequence using the 56 Env sites within 7.5Å of TMR. **(A)** Performance of the GB regressor model: MSE, Mean squared error; MAE, mean absolute error; RMSE, root mean squared error; RMAE, root mean absolute error. **(B)** SHAP values for each sequence feature across all 778 samples, capturing the magnitude, direction, and distribution of feature impact on model performance. Red and blue describe the presence or absence of the feature in each sample, respectively. Features with the highest SHAP values are shown, and all data are provided in **Data File S4**. Note that several sequence features shown represent the TMR-sensitive amino acid variant.

(C,D) Probabilistic model to detect low-prevalence mutations suspected of increasing resistance to TMR. **(C)** For all rare mutations at sites within 7.5Å of TMR (see text), we examined their frequency in the combined dataset of 778 samples. Frequencies are plotted against the average TMR resistance of the samples that contain them. Data points are colored by the variability in IC_{50} values. **(D)** Summary of the rare and potentially impactful features for the 19 mutations with the highest ranks (i.e., highest likelihood for resistance). The smallest distance between the closest atoms of the indicated position and TMR is shown. $\bar{x}$, average $\log_{10}$ TMR IC_{50} among the samples that contain the mutation; s, variability (in standard deviation) among the $\log_{10}$ IC_{50} values of the samples that contain the mutation.

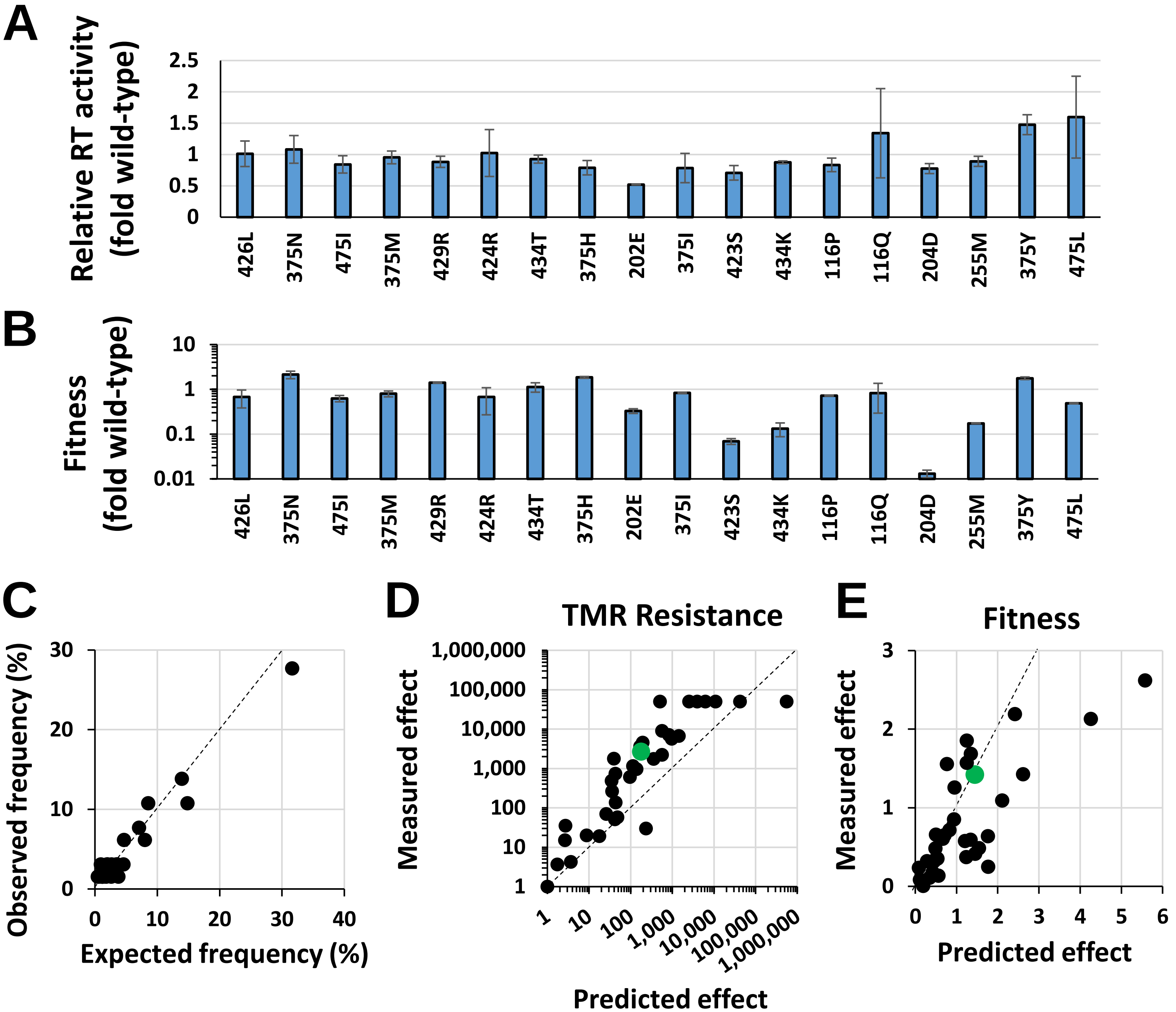

Supplemental Figure S5. Measurements of REM fitness and resistance to TMR. Related to Fig 3.

(A,B) Production efficiency and infectivity of REM-containing viruses. Viruses that contain the indicated REMs were produced and tested for infection of Cf2Th-CD4+CCR5+ cells. At least 3 independent batches of the virus were generated and tested. The virus content in each sample was measured by RT activity (in mU/mL). The level of RT activity in the samples (relative to the wild-type Env produced in the same batch) is shown in panel **A** (error bars, SEM). Fitness of the REMs from each batch was calculated as the ratio between infectivity and virus content. This value was then expressed as a fraction of that measured for the wild-type Env in the same experiment. The averages across the three batches are shown in panel **B** (error bars, SEM). REMs are ordered by their emergence frequency in the BRIGHTE escape group, with the highest-frequency REM on the left.

(C-E) Effects of mutation combinations on Env fitness and TMR resistance. **(C)** The expected frequency represents the product of the individual frequencies of the REMs in the escape group. The observed frequency describes the percent of individuals from the escape group that developed both mutations after treatment. **(D,E)** Synergy between the effects of mutations on Env resistance to TMR and fitness. The measured effect of two-mutation combinations on resistance or fitness (shown in **Fig 3F**) is compared with the predicted effect, which is calculated as the product of the effects of the individual mutations on the respective phenotype. The datapoint for the 375N/426L combination is shown in green. The dashed lines represent the lines of equivalence (x = y).

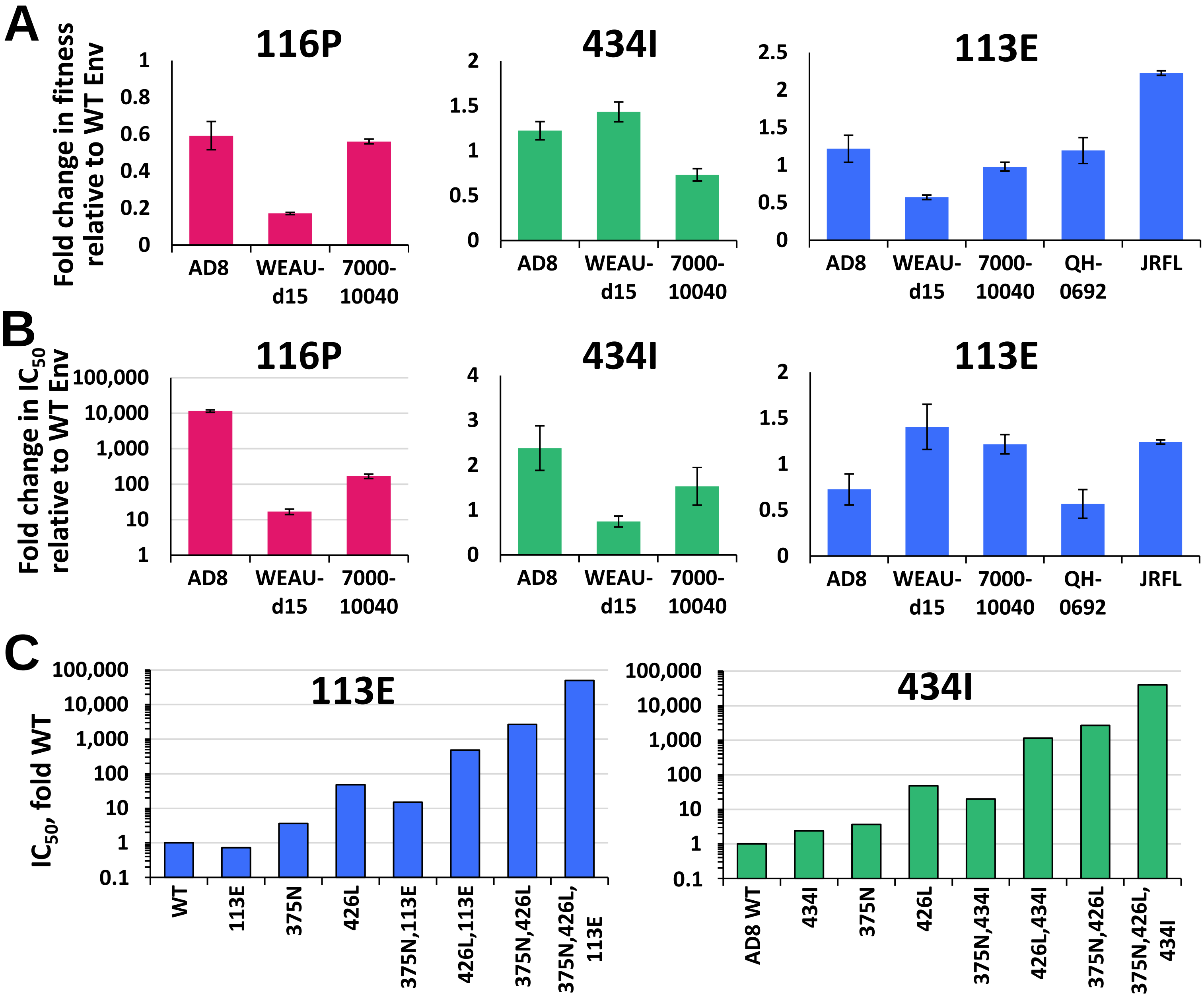

Supplemental Figure S6. Effects of mutations on Env fitness and TMR resistance in diverse isolates. Related to Fig 3.

(A,B) The indicated mutations were introduced in AD8 Env and in the T/F strains WEAUd15.410.5017 (accession number EU289202) and 700010040.C9.4520 (EU576418). Effects on Env fitness (**A**) and resistance to TMR (**B**) are expressed as their fold change relative to the corresponding wild-type Envs. Given the high emergence frequency of 113E after treatment, we also examined the effects of this mutation on strains QH-0692_42 (AY835439) and JRFL (U63632). Error bars, SEM.

(C) Enhancement of REM effects on TMR resistance by the 113E and 434I mutations. The fold increase in IC_{50} of virus containing the AD8 Env with the indicated mutations (relative to the wild-type form) is shown.

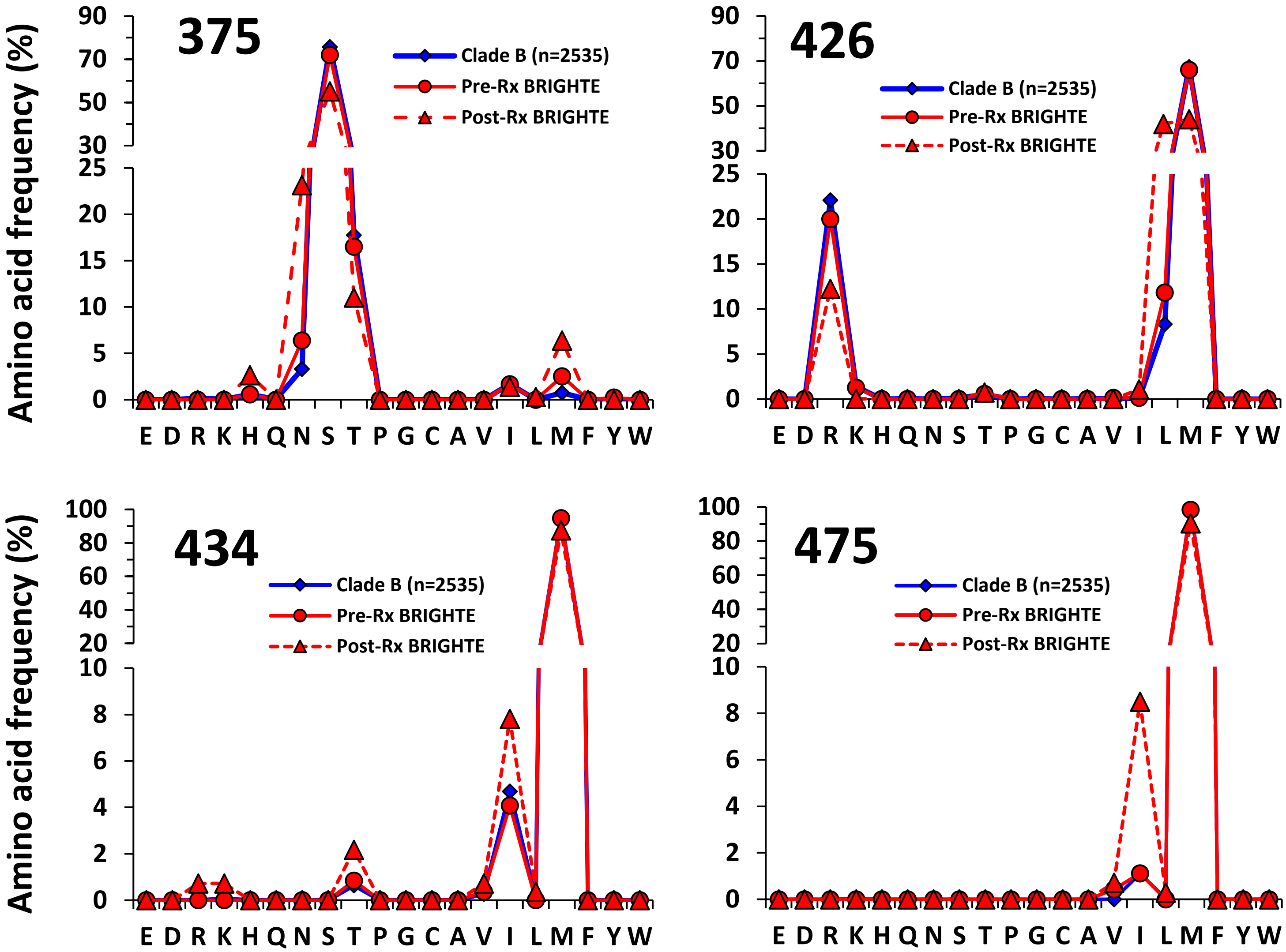

Supplemental Figure S7. Frequency distribution of all amino acids at Env positions associated with resistance to TMR. Related to Fig 4.

Frequencies are expressed as a percent of all variants at that position and are shown for: **(i)** The population of HIV-1 clade B isolates, represented by the panel of 2,535 Envs, **(ii)** Samples collected in the BRIGHTE trial before treatment, and **(iii)** Samples collected in the BRIGHTE trial after treatment.

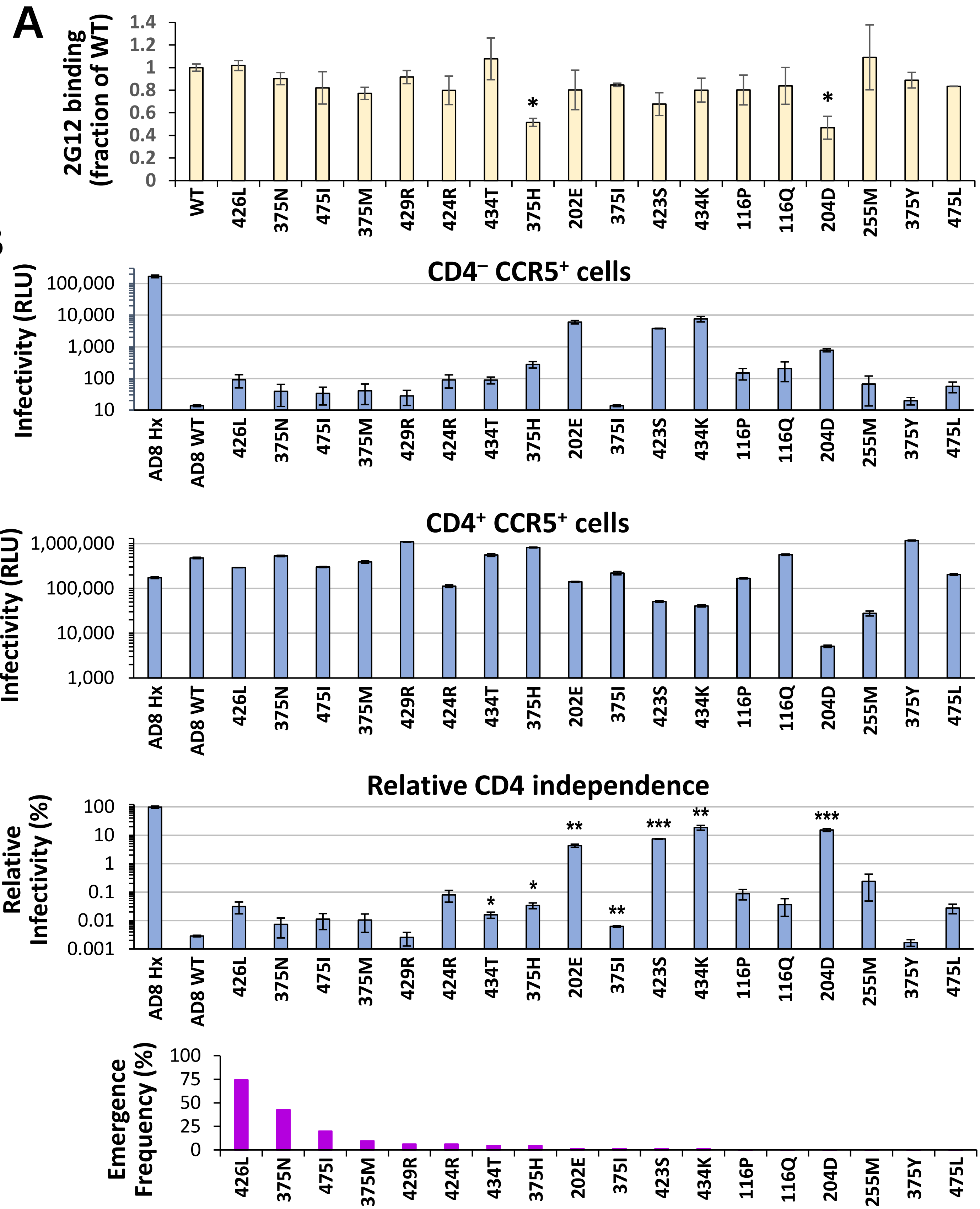

Supplemental Figure S8. Relative expression and CD4-independent infection of REMs. Related to Fig 5.

(A) Binding of the 2G12 antibody to HOS cells that express the REMS, as measured by cell-based ELISA**.**

(B) The indicated mutations were introduced in AD8 Env, and pseudoviruses were used to infect Cf2Th cells that stably express human CCR5 or both CD4 and CCR5. Relative CD4 independence is calculated as the level of infection measured in the CCR5+ cells as a percent of infection measured in the CD4+CCR5+ cells. The CD4-independent mutant AD8/Hx(N197S) was used as a positive control. REMs appear from left to right in decreasing order of frequency in the escape group. Error bars, SEM. P-values calculated in a two-tailed t-test relative to the wild-type Env: *, P<0.05; **, P<0.005; ***, P<0.0005. Emergence frequency of the REMS in the BRIGHTE escape group is shown for reference in the bottom panel.

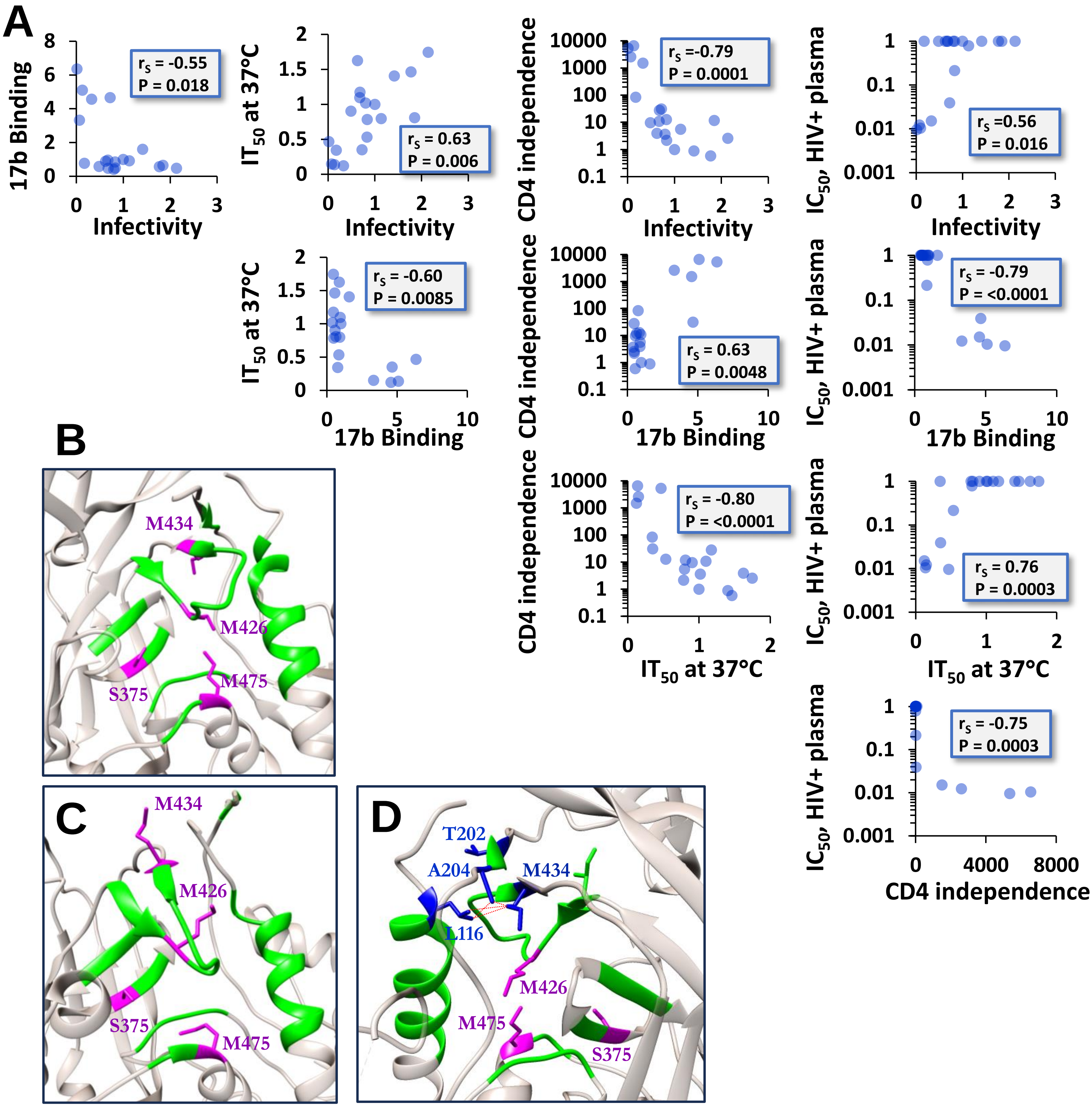

Supplemental Figure S9. Env features associated with REMs and proposed mechanism of TMR resistance. Related to Fig 5.

(A) Relationships between Env features we measured for the 18 REMs. All values describe the effects of the REMs relative to the wild-type AD8 Env. r_S, Spearman rank correlation coefficient; P-value, two-tailed test.

(B-D) Mechanism of Env resistance to TMR. **(B,C)** Arrangement of the SM^3 sites (in pink) in the unliganded **(B)** and CD4-bound **(C)** forms of the clade B Env B41 (PDB IDs 6U59 and 5VN3, respectively). **(D)** Clasp-like structure that we propose maintains Env in the native state, formed by L116, M434 and A204. Mutations at these positions increase exposure of the CoR-BS, enhance sensitivity to non-neutralizing plasma, reduce Env fusion competence and increase virus inactivation rate at 37°C. The SM^3 sites 375, 426 and 475 are shown for orientation as well as position 202, which is also associated with transition to a CD4-bound-like conformation.

A

From nucleotide (columns) / **To nucleotide** (rows)

To \ From	A	C	T	G
**A**		5.9	1.9	25.0
**C**	4.7		22.6	1.7
**T**	8.2	7.4		8.8
**G**	10.8	0.4	2.7	

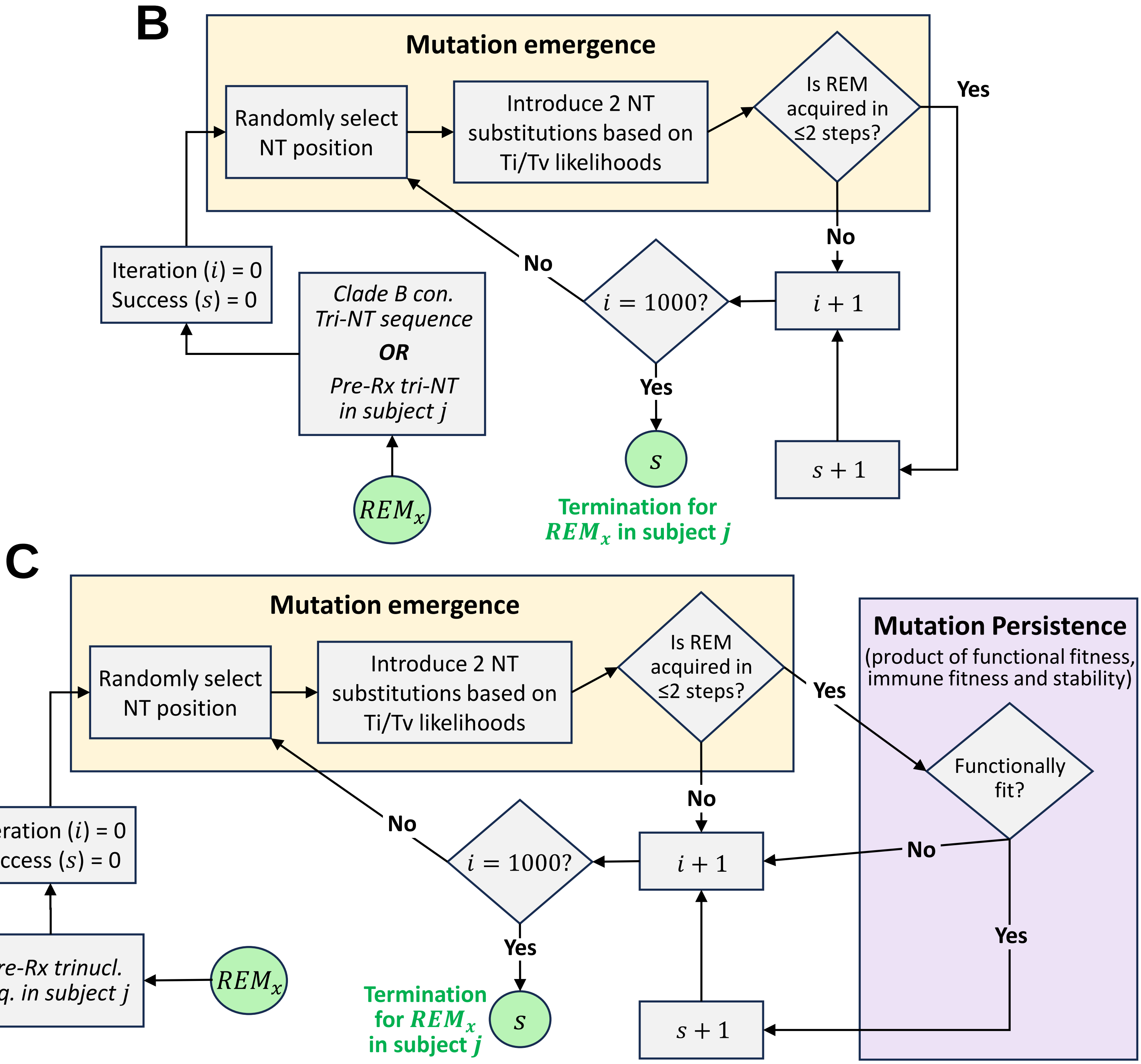

Figure S10. Simulation of the emergence and persistence of REMs in the population and in TMR-treated individuals. Related to Fig 6.

(A) Base substitution types detected in an acellular assay using reverse transcriptase of HIV-1 strain BH10, as described by Martínez del Río, *et al*., 2024. Values are expressed as a percent of all base substitutions detected.

(B) Simulation to determine the likelihood for appearance of REMs within two NT substitutions, using the clade B consensus sequence at each position or the pre-treatment sequences from each BRIGHTE participant. The number of success events (s) from the 1000 iterations (i) is calculated for each participant and averaged across the 65 subjects of the escape group.

(C) The above algorithm augmented with a decision module that accounts for the combined fitness of each REM.

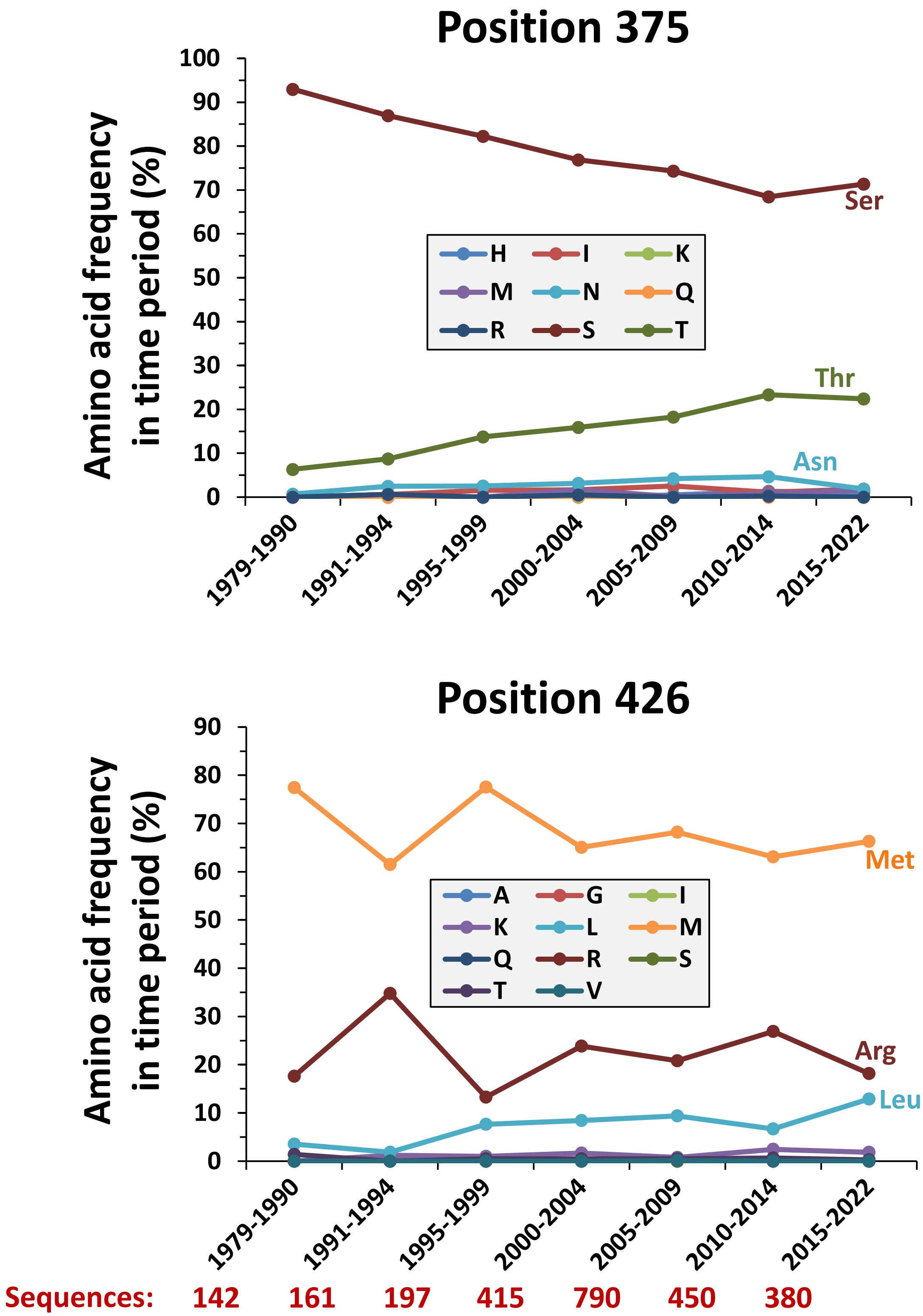

Supplemental Figure S11. Historical changes in amino acid occupancy at positions 375 and 426 in HIV-1 clade B. Related to Fig 6.

The 2535 sequences of the fostemsavir-untreated subjects infected by HIV-1 clade B were partitioned according to the year of sample collection. The frequency of all amino acids in each time period was calculated. The number of sequences in each time period is shown below the graphs in red.

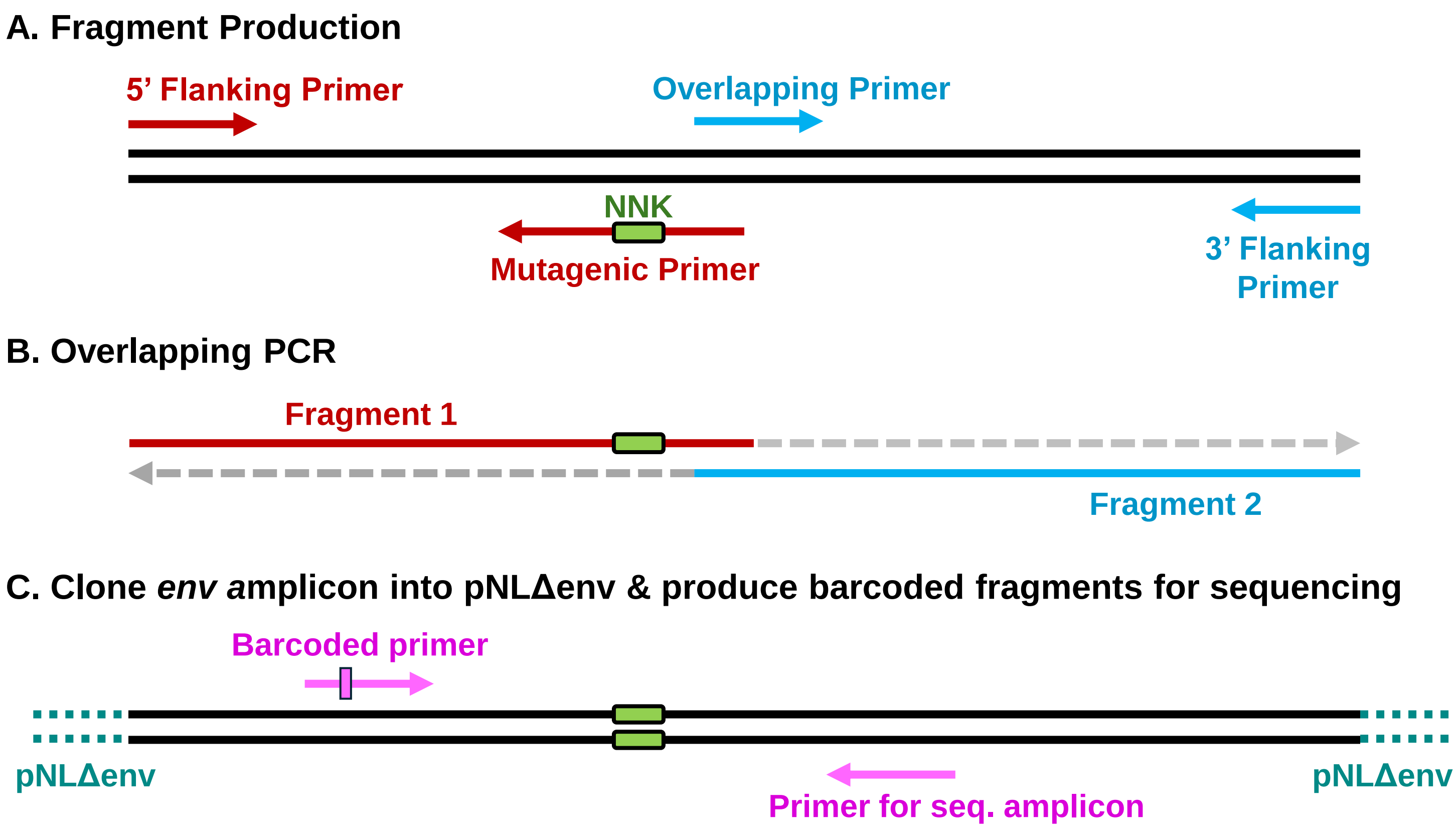

Supplementary Figure S12. Production of plasmid libraries for deep mutational scanning of positions 375 and 426. Related to STAR Methods.

Fragment 1 is produced by PCR using a universal 5′ flanking primer and a mutagenic primer containing the NNK codon. Fragment 2 is generated using an overlapping primer with 20–30 bp of homology downstream of the NNK site and a universal 3′ flanking primer. Fragments 1 and 2 are joined by overlap-extension PCR to yield the full-length amplicon that spans the *env* gene. The amplicon is then cloned into the env-deleted proviral vector pNL*Δ*env by the InFusion method. Barcoded primers were used to amplify the region containing the NNK site to allow multiplexing of samples in sequencing reactions.

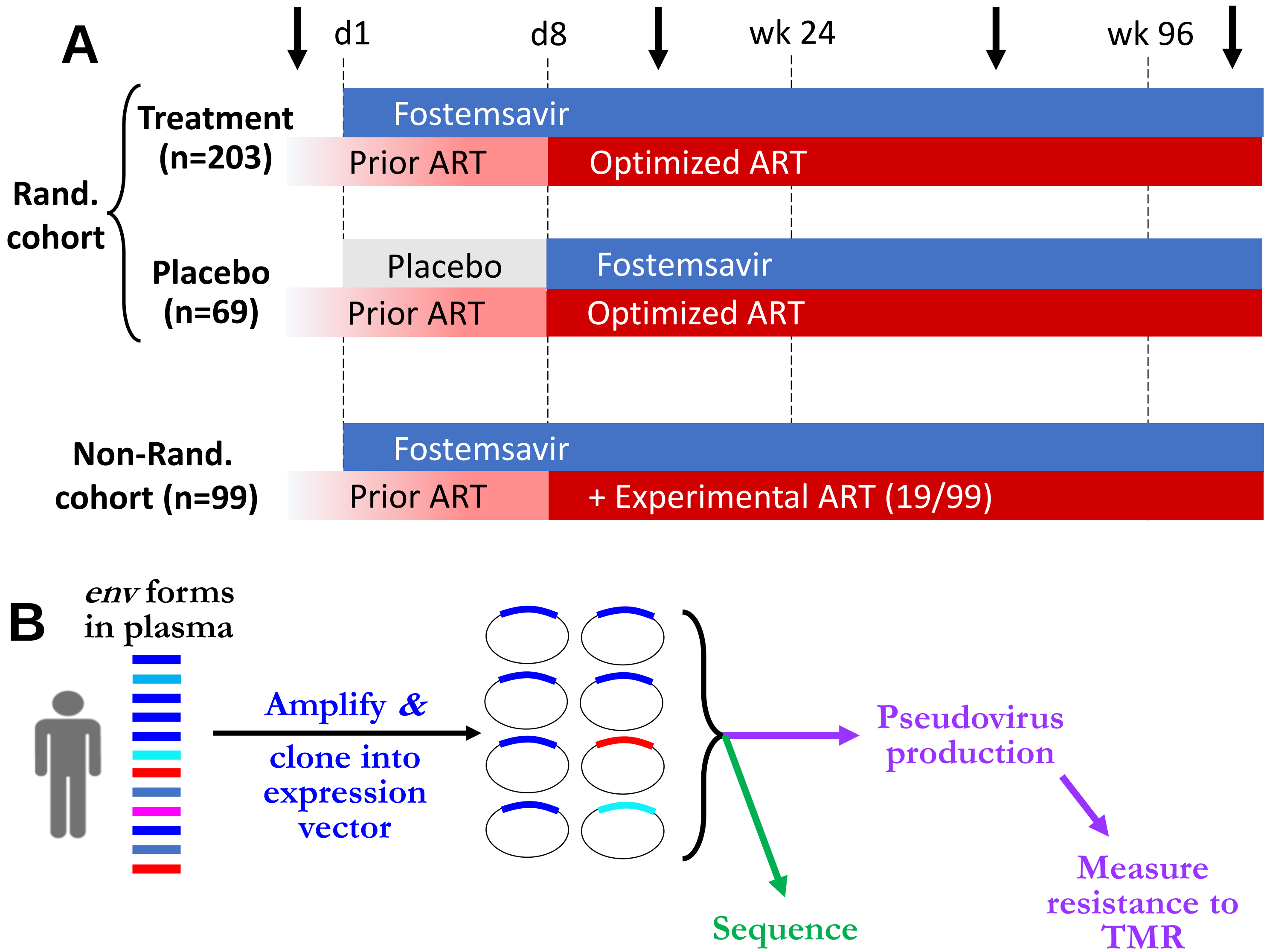

Subject	Day	1	2	3	4	…	856	IC_{50} (μM)
**1**	-46	M	R	A	K/R		L	**0.0004**
**1**	239	M	K	V/A	K		L	**0.6**
**…**								
**360**	-15	M	T	V	T/K		L	**0.03**
**360**	64	M	T	V	T		L	**>5**

Supplemental Figure S13. Design of the BRIGHTE clinical trial. Related to STAR Methods.

(A) Prior to trial initiation, phenotyping assays were conducted for plasma samples from all subjects to determine virus sensitivity to different ART classes. Based on these results, subjects for whom a complete ART regimen could not be constructed were assigned to the non-randomized cohort and all others to the randomized cohort. Fostemsavir (800 mg bid) or placebo was administered for 8 days, after which this regimen was continued and supplemented by an optimized ART regimen. At different time points before and during treatment, blood samples were collected (represented by arrows) and analyzed for viral load, CD4 count, and used for Env phenotyping and genotyping assays.

(B) The PhenoSense GT Entry Assay is based on cloning a library of amplicons containing the *env* gene into an expression vector. The plasmid library is used to generate a library of pseudoviruses that is tested *in vitro* for resistance to TMR. Samples were also sequenced using the HIV Envelope NGS Sequencing Assay. Given the in-host diversity of Env, many sites were assigned more than one amino acid, which we addressed by one-hot feature encoding, as described in the STAR Methods Section.

Supplemental Table S1. Env positions used as input for the XGBoost model to estimate resistance to TMR.

Env Position[a]	Distance (Å)[b]	Env atom[c]	TMR atom[d]
**113**	1.65	OD1	H23
**255**	2.33	O	H3
**432**	2.51	OE1	H21
**375**	2.52	OG	H6
**427**	2.70	CE2	H2
**433**	2.85	O	H19
**109**	2.88	O	H13
**424**	2.94	CG1	H18
**425**	3.00	O	H17
**112**	3.02	CZ2	H9
**202**	3.09	CG2	H19
**376**	3.17	N	H14
**434**	3.25	CE	H18
**475**	3.41	SD	H1
**370**	3.44	OE1	H5
**384**	3.50	OH	H5
**426**	3.60	N	H17
**116**	4.07	CD1	C29
**256**	4.19	N	H3
**108**	4.45	O	O09
**428**	4.68	N	O03
**383**	4.71	O	H6
**257**	4.73	N	H14
**382**	4.85	CZ	O06
**110**	4.94	N	H13
**200**	5.00	O	H19
**377**	5.05	N	H3
**117**	5.34	CA	H20
**114**	5.59	N	H22
**201**	5.70	C	H19
**479**	6.01	CZ2	H10
**69**	6.02	CZ2	H9
**374**	6.02	C	H14
**254**	6.02	C	H3
**111**	6.06	N	H13
**373**	6.19	O	H6
**435**	6.22	N	H15
**423**	6.32	C	H17
**105**	6.51	O	O09
**121**	6.55	CE	N32
**253**	6.60	O	H9
**371**	6.68	N	H6
**431**	6.69	O	H19
**369**	6.70	O	H6
**473**	6.80	O	H1
**478**	6.87	OD1	H3
**203**	6.93	N	H19
**421**	7.00	CE	H5
**429**	7.03	N	O03
**210**	7.16	CG	O06
**106**	7.21	O	H13
**199**	7.33	OG	H19
**115**	7.34	N	H22
**422**	7.49	O	H18
**107**	7.49	O	O09
**474**	7.49	C	H1

Env Position	Distance (Å)	Env atom	TMR atom
**368**	7.59	OD2	H5
**436**	7.68	N	H18
**118**	7.68	N	H20
**430**	7.73	O	N28
**204**	7.91	N	H19
**68**	7.97	CG2	H7
**258**	7.99	N	H14
**259**	8.17	O	H14
**261**	8.26	CD1	H14
**120**	8.30	O	H19
**385**	8.31	N	H6
**476**	8.39	N	H1
**378**	8.44	N	H3
**381**	8.48	O	C23
**119**	8.48	O	H20
**104**	8.56	O	O09
**380**	8.71	O	O06
**372**	8.87	N	H6
**419**	8.92	O	H6
**195**	9.12	OD1	H19
**122**	9.13	N	H19
**212**	9.20	CD	O06
**472**	9.55	CA	H14
**211**	9.61	N	O06
**420**	9.83	C	H6
**209**	9.86	O	H9
**471**	9.93	O	H14
**453**	10.13	CD1	H14
**437**	10.18	CD	H18
**205**	10.20	N	H19
**180**	10.24	OD1	H5
**260**	10.32	N	H14
**477**	10.50	N	H1
**179**	10.54	CD1	H6
**123**	10.56	CG2	N32
**252**	10.67	O	H9
**198**	10.69	OG1	H15
**67**	10.79	OD1	H9
**103**	10.88	O	O09
**379**	10.88	N	O06
**194**	11.04	CG2	H17
**387**	11.09	OG1	H14
**208**	11.26	CG2	H8
**418**	11.38	CB	H6
**386**	11.43	N	H6
**296**	11.67	SG	H14
**331**	11.72	SG	H14
**102**	11.74	O	O09
**213**	11.87	N	H9
**447**	12.02	OG	H3
**101**	12.08	O	H10
**445**	12.09	SG	H14
**196**	12.14	N	H19
**262**	12.15	ND2	H3
**294**	12.38	CD1	H14
**367**	12.38	C	H6
**217**	12.42	OH	O09

[a] Positions located within 4Å, 5Å, 7.5Å, 10Å and 12.5Å are highlighted in different shades of blue.

[b] The minimal distance between each Env position and any TMR atom was calculated using coordinates of the TMR-bound structure (PDB ID 5U7O).

[c,d] The Env and TMR atoms involved in the interactions.

Supplemental Table S2. Structure-guided approach to identify mutations that may increase Env resistance to TMR.

Position [a]	109	112	113	202	255	375	424	426	427	432	434	116	257	370	382	384	475
Distance from TMR [b]	2.9	3.0	1.7	3.1	2.3	2.5	2.9	3.6	2.7	2.5	3.3	4.1	4.7	3.4	4.9	3.5	3.4
Interacts with TMR? [c]	Yes	Yes	Yes	Yes	Yes	Yes	Yes	Yes	Yes	Yes	Yes	No	No	No	No	No	No
**Ala** [d]	0	0	0.1	0.1	0.6	0	0.04	0.04	0	0	0	0	0.04	0.1	0	0	0
**Arg**	0	0	0	1.2	0	0.2	0.04	22.1	0.2	7.4	0	0	0	0	0	0	0
**Asn**	0	0	0.1	0.04	0	3.3	0	0	0	0.1	0	0	0.04	0.04	0	0	0
**Asp**	0	0	98.2	0	0	0	0	0	0	0	0	0	0	0.04	0	0	0
**Cys**	0	0	0	0	0	0	0	0	0.12	0	0	0	0	0	0	0	0
**Glu**	0	0	1.7	0	0	0	0	0	0	0.1	0	0.04	0	99.8	0	0	0
**Gln**	0	0	0	0.04	0	0.04	0	0.04	0	0.7	0	0	0	0.04	0	0	0
**Gly**	0	0.04	0	0	0	0	0	0.04	0.12	0	0	0	0	0	0	0	0
**His**	0	0	0	0	0	0.6	0	0	0	0	0	0	0	0	0	0	0
**Ile**	98.3	0	0	0.04	2.0	1.7	78.8	0.08	0	0.1	4.7	0.3	0.04	0	0	0	1.1
**Leu**	0.04	0	0	0	0.04	0	0	8.3	0	0	0.1	99.6	0.04	0	0	0	0.04
**Lys**	0.4	0	0	4.5	0	0.04	0	1.4	0	91.2	0.1	0	0	0.04	0	0	0.04
**Met**	0	0	0	0	0.1	0.8	0.04	67.4	0	0	94.2	0.04	0	0	0	0	98.8
**Phe**	0	0	0	0	0	0	0	0	0	0	0	0.04	0	0	99.9	2.1	0
**Pro**	0	0	0	0	0	0	0	0	0	0	0	0	0.1	0	0	0	0
**Ser**	0.1	0	0	0.2	0.04	75.7	0	0.1	0	0	0	0	0.04	0	0	0	0
**Thr**	0.2	0	0	94.0	0	17.7	0	0.5	0	0.4	0.6	0	99.7	0	0	0	0
**Trp**	0	100.0	0	0	0	0	0	0	99.6	0	0	0	0	0	0	0	0
**Tyr**	0	0	0	0	0	0	0.04	0	0	0	0	0	0	0	0.1	98.0	0
**Val**	1.0	0	0	0	97.3	0	21.1	0.04	0	0.04	0.3	0.04	0	0	0	0	0

[a] Env positions with side chains that occupy the CD4-binding pocket and are within 5Å of the TMR molecule were identified using the TMR-liganded structure of Env (PDB ID 5U7O).

[b] Distance (in Å) between the closest atoms of the TMR molecule and the residue found at the indicated position on the structure.

[c] Absence or presence of an interaction between the side chain of the indicated amino acid and TMR, as suggested by the above structure.

[d] The frequency of all amino acids at the indicated positions was calculated using a panel of 2,535 isolates from HIV-1 clade B. Residues with a frequency higher than 0.5% at each site were further analyzed for their effects on Env fitness and resistance to TMR.

Supplemental Table S3. Summary of all mutations identified by the four approaches as suspected of increasing Env resistance to TMR.

Position	AA in con. B	GB Regressor[a]	Probabilistic model[b]	Structure guided[c,d]	Previously published	
					Mutation[e]	Reference
109	Ile			Val		
113	Asp	Glu	Ala, Glu, His	Glu, Gly		
116	Leu				Pro, Gln	Zhou et al, 2014
200	Val			Ser		
202	Thr			Ala, Lys, Ser, Arg	Glu	Gartland et al., 2024
204	Ala				Asp	Zhou et al, 2014
210	Phe	Trp				
255	Val	Ile	Ala	Ala, Ile, Met		
373	Met	Thr	Gln			
375	Ser	Thr, His, Asn	His, Met, Tyr, Ile	Asn, His, Ile, Met, Thr	His, Met, Thr	Pancera et al, 2017, Zhou et al, 2014
376	Phe		Ile, Leu			
377	Asn		Val			
384	Tyr		Leu	Phe, Cys		
423	Ile		Tyr		Tyr, Phe, Ser	Zhou et al, 2010
424	Ile	Val	Arg	Val		
426	Met	Leu	Ile	Arg, Leu, Lys, Thr	Leu	Zhou et al, 2014, Ray et al, 2013
429	Glu	Arg	His			
432	Lys	Gln	Leu	Arg, Gln	Leu	Pancera et al, 2017
434	Met	Lys, Ile		Ile, Thr, Leu	Ile, Lys	Zhou et al, 2014, Lataillade et al, 2018
475	Met	Ile		Ile, Leu	Ile	Pancera et al, 2017; Zhou et al, 2014
478	Asn		Ile			
506	Val				Met	Ray et al, 2013
595	Ile				Phe, Val	Zhou et al, 2010
655	Lys				Glu, Ala	Zhou et al, 2010

[a] The 14 mutations with the highest SHAP values are indicated.

[b] Mutations identified by the probabilistic model.

[c] All Env positions with side chains that occupy the CD4-binding pocket and are within 5Å of the TMR molecule were analyzed for the frequency of each amino acid in the panel of 2,535 clade B Envs. All residues with a frequency of 0.5% or higher are shown. See all data in Table S2.

[d] Mutations in red font were selected based on the structure, but their prevalence in the population was lower than 0.5%.

[e] Mutations in red font were not explicitly noted in the references but were added due to suspected effects.
